# Ceftriaxone exerts antitumor effects in *MYCN*‐driven retinoblastoma and neuroblastoma by targeting DDX3X for translation repression

**DOI:** 10.1002/1878-0261.13553

**Published:** 2023-11-27

**Authors:** Pamorn Chittavanich, Duangporn Saengwimol, Sittiruk Roytrakul, Duangnate Rojanaporn, Vijender Chaitankar, Atthapol Srimongkol, Usanarat Anurathapan, Suradej Hongeng, Rossukon Kaewkhaw

**Affiliations:** ^1^ Program in Translational Medicine, Faculty of Medicine Ramathibodi Hospital Mahidol University Bangkok Thailand; ^2^ Research Center, Faculty of Medicine Ramathibodi Hospital Mahidol University Bangkok Thailand; ^3^ Functional Proteomics Technology Laboratory, National Center for Genetic Engineering and Biotechnology National Science and Technology Development Agency Pathum Thani Thailand; ^4^ Department of Ophthalmology, Faculty of Medicine Ramathibodi Hospital Mahidol University Bangkok Thailand; ^5^ Biodata Mining and Discovery Section, National Institute of Arthritis and Musculoskeletal and Skin Diseases National Institutes of Health Bethesda MD USA; ^6^ Department of Pediatrics, Faculty of Medicine Ramathibodi Hospital Mahidol University Bangkok Thailand; ^7^ Chakri Naruebodindra Medical Institute, Faculty of Medicine Ramathibodi Hospital Mahidol University Samut Prakan Thailand

**Keywords:** ceftriaxone, DDX3X, *MYCN* amplification, neuroblastoma, retinoblastoma, translation

## Abstract

MYCN proto‐oncogene, bHLH transcription factor (*MYCN*) amplification is associated with aggressive retinoblastoma (RB) and neuroblastoma (NB) cancer recurrence that is resistant to chemotherapies. Therefore, there is an urgent need to identify new therapeutic tools. This study aimed to evaluate the potential repurposing of ceftriaxone for the treatment of *MYCN*‐amplified RB and NB, based on the clinical observations that the drug was serendipitously found to decrease the volume of the *MYCN*‐driven RB subtype. Using patient‐derived tumor organoids and tumor cell lines, we demonstrated that ceftriaxone is a potent and selective growth inhibitor targeting *MYCN*‐driven RB and NB cells. Profiling of drug‐induced transcriptomic changes, cell‐cycle progression, and apoptotic death indicated cell‐cycle arrest and death of drug‐treated *MYCN*‐amplified tumor cells. Drug target identification, using an affinity‐based proteomic and molecular docking approach, and functional studies of the target proteins revealed that ceftriaxone targeted DEAD‐box helicase 3 X‐linked (DDX3X), thereby inhibiting translation in *MYCN*‐amplified tumors but not in *MYCN‐*nonamplified cells. The data suggest the feasibility of repurposing ceftriaxone as an anticancer drug and provide insights into the mechanism of drug action, highlighting *DDX3X* as a potential target for treating *MYCN*‐driven tumors.

## Graphical abstract

The study sought to repurpose ceftriaxone for *MYCN‐*amplified retinoblastoma (RB) and neuroblastoma (NB) treatment due to its observed capability to reduce *MYCN*‐driven RB subtype volume. Patient‐derived tumor organoids and cell line models demonstrated ceftriaxone's selectivity in targeting *MYCN*‐driven cancer cells through DDX3X inhibition, suppressing translation. This identifies ceftriaxone's potential as an anticancer agent, highlighting DDX3X as a treatment target.

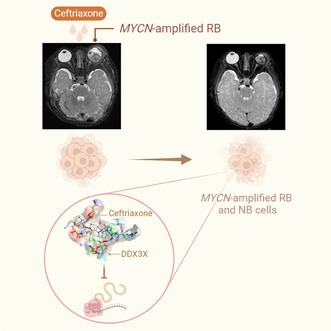

**Abbreviations**
ATP: adenosine triphosphate
DDX3X: ATPase‐dependent DEAD‐box RNA helicase
DEGs: differentially expressed genes
DREAM: dimerization partner (DP), retinoblastoma (Rb)‐like, E2F, and MuvB
DTI: drug–target interaction
*E*_max_
: fraction of viable cells at the highest drug concentration
GO: Gene Ontology
GSEA: gene set enrichment analysis
HRP: horseradish peroxidase
IC_50_
: half‐maximal inhibitory concentration
KEGG: Kyoto Encyclopedia of Genes and Genomes
LC–MS/MS: liquid chromatography–tandem mass spectrometry
l‐HPG: l‐homopropargylglycine
MYCN: MYCN proto‐oncogene, BHLH transcription factor
NADH: reduced nicotinamide adenine dinucleotide
NB: neuroblastoma
NDUFA9: NADH:ubiquinone oxidoreductase subunit A9
NES: normalized enrichment score
NGFR: nerve growth factor receptor
OXPHOS: oxidative phosphorylation
PBMCs: peripheral blood mononuclear cells
RB: retinoblastoma
TAMRA: tetramethylrhodamine

## 1 Introduction

*MYCN* amplification is the most common genetic aberration associated with poor prognosis in neuroblastoma (NB) [1, 2, 3] and is identified as the primary driver in a rare and aggressive subtype of retinoblastoma (RB) [4, 5]. Compared with the classical *RB1*‐deficient RB, *MYCN*‐amplified RB has histopathological and genomic characteristics more similar to NB with *MYCN* amplification [4, 6]. These *MYCN*‐amplified tumors have distinct gene signatures associated with the resultant MYCN overexpression, retaining an undifferentiated and aggressive phenotype [7, 8, 9, 10]. Given these similarities, the RB and NB with *MYCN* amplification may display similar responses to the same drug regimen. However, current chemotherapies are ineffective and fail to treat the *MYCN*‐amplified RB and NB with aggressive recurrence [1, 11]. Thus, there is a high demand for novel and effective therapies for both tumors.

Drug repurposing provides opportunities for developing drug molecules with new therapeutic indications, especially for *MYCN*‐amplified NB and RB cancers of high unmet need [12, 13, 14]. Candidate drugs with known safety profiles could gain high approval rates for clinical use. Additionally, the investigation of off‐target molecules of existing drugs leads to the discovery of previously unrecognized protein targets, which is expected to contribute to rational drug design for new indications [15]. Interestingly, we serendipitously found in this study that ceftriaxone, an FDA‐approved third‐generation cephalosporin antibiotic, could decrease the volume of unexpected RB with *MYCN* amplification subtype in a patient initially diagnosed with orbital cellulitis. However, the drug effects observed in the patient are not pertinent to the inhibition of the penicillin‐binding proteins, the drug targets that are not produced in eukaryotic cells; thus, an off‐target drug activity might be responsible for the pleiotropic effects of ceftriaxone in tumors.

Supporting evidence for the antigrowth effect of ceftriaxone in the xenograft models of lung cancers has suggested that the drug exhibits polypharmacology, having activities beyond anti‐infections [16]. However, the drug effects and mechanism of action for ceftriaxone are unknown in *MYCN*‐amplified RB cells; whether this drug has therapeutic effects in NB with *MYCN* amplification remains to be elucidated.

This study aimed to evaluate the potential antitumor activity of ceftriaxone and identify the target molecules of ceftriaxone in *MYCN*‐amplified RB and NB. Here, we demonstrated ceftriaxone activities in patient‐derived RB organoids and NB cell lines. Drug targets were identified by examining drug‐associated gene signatures and drug‐protein interaction networks to illustrate the underlying mechanisms of action for ceftriaxone. Our study indicates the feasibility of ceftriaxone repurposing and reveals previously unrecognized drug targets, which merit rational drug design for *MYCN*‐amplified tumors and future studies of these cancers.

## 2 Materials and methods

### 2.1 Tumor organoids and cell lines

Retinoblastoma organoids, RB170 (*MYCN* amplification) and RB668, RB654, and RB394 (*RB1*−/−), were previously established at Ramathibodi Hospital (December 2015 to November 2018) [14, 17, 18]. Matrigel‐embedded organoids were maintained in neurobasal medium (Gibco, Thermo Fisher Scientific, MA, USA), supplemented with 20 pg·mL^−1^ EGF, 10 pg·mL^−1^ bFGF, 1× B27 (Gibco), 2.5% knockout serum replacement (Gibco), and 2.5% fetal bovine serum (FBS), and passaged, as described previously [18]. The RB cell line (Y79; RRID: CVCL_1893), *MYCN*‐amplified NB cell lines (IMR32; RRID: CVCL_0346 and SK‐N‐BE (2); RRID: CVCL_0528), and *MYCN*‐nonamplified NB cell lines (SH‐SY5Y; RRID: CVCL_0019, SK‐N‐SH; RRID: CVCL_0531, and SK‐N‐AS; RRID: CVCL_1700) were purchased from American Type Culture Collection (ATCC, Manassas, VA, USA) and maintained in accordance with the manufacturer's instructions. Cell lines were maintained in RPMI‐1640 (Y79), MEM alpha modification (IMR32), DMEM/F12 (SK‐N‐BE (2), SH‐SY5Y, SK‐N‐SH), or DMEM/F12 high glucose (SK‐N‐AS) (all from HyClone, Logan, UT, USA) supplemented with 10% FBS. The authentication process for organoid lines is detailed in [14, 18]. All cell lines underwent regular mycoplasma screening, and all experiments were conducted using mycoplasma‐free cells.

### 2.2 Cell viability assay

The cell viability after ceftriaxone (Rocephin) treatment was evaluated using the CellTiter‐Glo luminescent cell viability assay (Promega, Madison, WI, USA). Subsequently, these data were utilized to generate dose–response curves, which were then fitted using nonlinear least squares regression with a four‐parameter variable slope model. These resultant curves were used to compute the half‐maximal inhibitory concentration (IC_50_), and the fraction of viable cells at the highest drug concentration (*E*_max_) using dr fit software [19].

### 2.3 Drug toxicity assay

Peripheral blood mononuclear cells (PBMCs) were collected at Ramathibodi Hospital (October 2023) and used to assess drug toxicity. Briefly, 10 mL of whole blood was mixed with anticoagulant citrate dextrose solution and then diluted in a 1 : 1 ratio with PBS containing 2% FBS. This mixture was subsequently layered over Ficoll Paque Plus (GE Healthcare, Chicago, IL, USA) and centrifuged at 400 ***g*** for 30 min. Subsequently, PBMCs were collected, washed with PBS, cultured in StemSpan™ SFEM (STEMCELL Technologies, Vancouver, Canada), and supplemented with 50 μg·mL^−1^ of stem cell factor, FMS‐like tyrosine kinase 3 ligand, interleukin‐3, and interleukin‐6 (all from PeproTech, Cranbury, NJ, USA). For cell viability and cell counting assays, PBMCs were seeded at densities of 2 × 10^4^ or 1 × 10^5^ cells and treated with specified ceftriaxone concentrations for 48 h. Cell viability was determined using the CellTiter‐Glo assay, and these data were subsequently used to construct dose–response curves. Viable cells, identified by their negative trypan blue staining, were counted using a hemocytometer.

### 2.4 Colony formation assay

Soft agar colony formation assays were performed as described previously [17]. Briefly, RB170 cells (10 000 cells/well of a 12‐well plate) were mixed with 0.35% of a low‐melting agarose gel (Sigma‐Aldrich, St.Louis, MO, USA) with drug or vehicle, plated on the bottom layer of a 0.5% agarose gel, and cultured for 3 weeks. NB cells (600 cells/well of a 12‐well plate) were incubated with a drug or vehicle for 2 weeks. Cultures were stained with 0.05% or 0.5% crystal violet in 40% methanol. The images were captured using a Chemidoc MP imaging system (Bio‐Rad, Hercules, CA, USA), and colonies were counted using imagej with the Cell Counter plugin (Fiji). Dose–response curves from the colony counts were constructed to compute IC_50_ values (graphpad v8, GraphPad Software Inc., La Jolla, CA, USA).

### 2.5 Cell‐cycle and apoptosis assays

Dissociated cells were stained with propidium iodide (PI) solution for cell‐cycle assays and with FITC Annexin V (BioLegend, San Diego, CA, USA) and PI for apoptosis assays. Data acquisition was performed using a BD FACSVerse flow cytometer (BD Biosciences, Franklin Lakes, NJ, USA), and the data were analyzed using flowjo software (BD Biosciences).

### 2.6 Mitochondrial ATP detection assay

Iodoacetamide (IAA), a GAPDH inhibitor, was used to inhibit glycolysis and, consequently, glycolytic ATP production. Simultaneously, the presence of pyruvate, a cell‐permeable substrate for mitochondrial Complex I, in the culture medium ensured the maintenance of ATP production from mitochondrial activity. Cells were exposed to ceftriaxone or a vehicle control in a medium supplemented with 1 mm pyruvate and 1 mm IAA for 1 h before conducting ATP detection via the CellTiter‐Glo Assay. Total ATP was detected in cancer cells cultured in the medium supplemented with pyruvate and without IAA. Subsequently, the luminescence readout (RLU) was converted into ATP concentration using an ATP standard curve.

### 2.7 Immunofluorescence

The cells were fixed with 4% paraformaldehyde for 15 min, whereas whole‐mount organoid samples were fixed using a mixture of 0.2% glutaraldehyde and 4% paraformaldehyde for 20 min. Samples were then permeabilized using 0.1% Triton X‐100 and blocked in a buffer solution (5% goat serum, 1% BSA, 0.05% Triton X‐100, pH 7.4). The fixed cells were then subjected to overnight incubation with a mouse anti‐Ki67 antibody (1 : 100, #550609; BD Pharmingen™, San Diego, CA, USA), followed by staining with Alexa Fluor 488 Goat anti‐Mouse IgG (1 : 500; Invitrogen, Carlsbad, CA, USA) for 1.5 h. Whole‐mount actin staining of organoids was conducted using Alexa Fluor 568 phalloidin staining (1 : 40, #A12380; Invitrogen). Nuclei were counter‐stained with DAPI (1 μg·mL^−1^). Ki67 staining was imaged using a Zeiss fluorescent microscope (Carl Zeiss AG, Oberkochen, Germany). Whole‐mount actin staining was imaged using confocal laser scanning microscopy, and a maximum intensity projection (Z‐stacking) was performed with nis‐element ar software (Nikon, Minato, Japan).

### 2.8 Western blotting

Cell lysates were extracted using radioimmunoprecipitation (RIPA) lysis buffer containing 1× Halt protease and phosphatase inhibitor cocktail (Thermo Fisher Scientific, Waltham, MA, USA). Briefly, 10–50 μg total protein was mixed with Laemmli sample buffer (Bio‐Rad), boiled, separated by 7.5% or 10% resolving polyacrylamide gel by SDS/PAGE with a 5% stacking gel, and transferred to polyvinylidene difluoride (PVDF) membranes. The membranes were blocked with 5% nonfat dry milk powder in tris‐buffered saline Tween‐20 (TBST) and incubated overnight at 4 °C with primary antibody [anti‐MYCN (1 : 500, sc‐56729; Santa Cruz, Dallas, TX, USA), anti‐c‐MYC (1 : 1000, #9402), anti‐MYCN/c‐MYC (1 : 1000, #13987), anti‐Aurora A (1 : 1000, #4718), anti‐Aurora B (1 : 1000, #3094), anti‐DDX3X (1 : 1000, #2635) all from Cell Signaling Technology, Danvers, MA, USA]. Membranes were washed with TBST, further incubated with an HRP‐conjugated secondary antibody at room temperature for 1 h, and washed with TBST. The target proteins were detected using a chemiluminescent HRP detection reagent (Millipore, Burlington, MA, USA). The membranes were probed with anti‐GAPDH or anti‐β‐actin HRP‐conjugated antibodies to ascertain a housekeeping loading control protein. The target proteins were detected using a chemiluminescent HRP detection reagent (Millipore), and the signal intensity was quantified with image lab software (Bio‐Rad). Relative densitometry of protein bands was expressed in relation to GAPDH or actin band densitometry and reported.

### 2.9 Reverse transcription‐quantitative real‐time PCR

Total RNA was extracted using TRIzol reagent (Invitrogen) in accordance with the manufacturer's instructions. Briefly, chloroform was added to samples treated with TRIzol reagent to generate phase separation. The aqueous phase, containing RNA, was then isolated for subsequent isopropanol precipitation. The RNA pellet was then washed and resuspended in RNase‐free distilled water. Total RNA (1 μg) was then reverse‐transcribed into cDNA using an Improm‐II reverse transcriptase system and random primers (Promega). Reverse transcription‐quantitative real‐time PCR (RT‐qPCR) was performed using SYBR green master mix (Bio‐Rad) on CFX96 Touch Real‐Time PCR Detection System (Bio‐Rad) with the thermal cycling condition as follows: initial denaturation (95 °C for 20 s) and 40 cycles of two‐step thermal profile (95 °C for 5 s and 60 °C for 30 s), followed by melting curve analysis to verify that the fluorescence detected during the run originates from a single amplicon. Target gene expression levels were normalized to β‐actin expression levels. Primer sequences were listed as follows: MYCN_E2_F: ACCACAAGGCCCTCAGTAC, MYCN_E3_R: TCGTTTGAGGATCAGCTCGC, Human β‐actin_F: GGCACCCAGCACAATGAAGATC, and Human β‐actin_R: GTAACGCAACTAAGTCATAGTCCGC.

### 2.10 Ceftriaxone‐conjugated sepharose 4B synthesis and isolation of ceftriaxone‐binding proteins

Briefly, 20 mg of cyanogen bromide‐activated‐Sepharose^®^ 4B (Sigma‐Aldrich) was coupled with 12.7 mg of ceftriaxone in the coupling buffer (0.1 m NaHCO_3_ and 0.5 m NaCl pH 8.5). Next, the beads were washed four times with the coupling buffer, followed by acetic buffers (0.1 m acetic acid, 0.5 m NaCl pH 4), and were subsequently stored in 1 m NaCl at 4 °C. Protein lysates (800 μg of input lysate) were incubated with the ceftriaxone‐conjugated beads (4 nmol of ceftriaxone in 20 μL) overnight at 4 °C with rotation. Following this incubation, the samples underwent three washes using the washing buffer, comprised of 50 mm Tris (pH 7.5), 5 mm EDTA, 150 mm NaCl, 0.01% NP‐40, and 0.02 mm phenylmethylsulfonyl fluoride. An additional wash was then performed using 0.01% NP‐40. Subsequently, the proteins were eluted with Laemmli sample buffer and boiled for western blotting. Alternatively, they were eluted with 0.5% SDS and further diluted to achieve a final concentration of 0.1% SDS for liquid chromatography–tandem mass spectrometry (LC–MS/MS). For the drug competitor assay, the lysate was preincubated with 200 mm ceftriaxone at 4 °C for 1 h before being incubated with ceftriaxone‐conjugated beads.

### 2.11 Sample preparation and LC–MS/MS

The disulfide bonds in the protein samples were reduced using 10 mm dithiothreitol in 10 mm NH_4_(HCO_3_) and subsequently blocked with 30 mm iodoacetamide in 10 mm NH_4_(HCO_3_). The proteins were then digested with trypsin at a 1 : 20 ratio for 16 h at 37 °C. Tryptic peptides were dried using a speed vacuum concentrator and resuspended in 0.1% formic acid for further analyses. For LC–MS/MS analysis, the digested peptide samples (5 μL) were enriched using a μ‐Precolumn 300 μm I.D. × 5 mm PepMap C18, 100 Å, 5 μm (Thermo Fisher Scientific) and separated on a Reverse‐Phase HPLC column (Acclaim PepMap RSLC C18, 100 Å, 2 μm, 75 μm I.D. × 15 cm, nanoViper; Thermo Fisher Scientific). The temperature during peptide separation was maintained at 60 °C by thermostatted column oven. A mobile phase solvent A (0.1% formic acid) and solvent B (0.1% formic acid in 80% acetonitrile) were utilized in the analytical column. Peptides were eluted using a 5–55% gradient of solvent B with a constant flow rate of 300 nL·min^−1^ for 30 min. The electrospray of eluted peptides was generated through CaptiveSpray at 1.6 kV and dried using nitrogen at a flow rate of approximately 50 L·h^−1^ to eliminate the solvent in the samples. Collision‐induced‐dissociation, using nitrogen gas as the collision gas, was performed to produce product ions. Both MS and MS/MS spectra were obtained in positive‐ion mode at 2 Hz over the range (*m*/*z*) 150–2200. The collision energy was adjusted to 10 eV as a function of the *m*/*z* value. The LC–MS/MS analysis of each sample was done in triplicate.

### 2.12 Analysis of proteomic data and drug–target interaction networks

The LC–MS/MS data were analyzed based on the peptide ion's signal intensities in MS mode using the decyder MS Differential Analysis software (GE Healthcare). The analyzed MS/MS data were then subjected to a database search using mascot software (Matrix Science, London, UK) for protein identification. The data were searched against the *Homo sapiens* protein database from UniProt (December 2021) for protein identification. This search allowed for a maximum of three missed cleavages, with carbamidomethylation of Cysteine as a fixed modification and oxidation of Methionine as variable modifications. Data visualization and statistical analyses were performed utilizing the Multiple Experiment Viewer (MeV) within the tm4 suite software (J. Craig Venter Institute, La Jolla, CA, USA).

The proteins identified from the samples with drug competitor added were subtracted as nonspecific protein‐drug binding, and proteins with confidence (protein ID score) cutoff set to > mean ID score were defined as drug‐binding proteins. To generate drug–target interaction (DTI) networks, drug‐binding proteins were analyzed using the plug‐in STRING with confidence (a score—mode for physical interactions) cutoff set to > 0.7 and clusterMaker Cluster Network (MCL Cluster) Apps in cytoscape 3.9.0 (Cytoscape Consortium). The networks from different conditions of ceftriaxone treatment were merged to create final DTI networks. Each DPI network was analyzed using STRING enrichment with an FDR value cutoff of 0.05 to enrich the relevant pathways (WikiPathways and KEGG Pathways) and Gene Ontology (GO) terms associated with biological processes, molecular functions, and cellular locations.

### 2.13 Translation and nascent MYCN detection assays

Tumor cells were exposed to an amino acid analog of methionine, l‐homopropargylglycine (l‐HPG), for 1 h in methionine‐free RPMI 1640 (Gibco). After treatment, cells were either lysed or fixed, and the nascent proteins labeled with l‐HPG were captured using TAMRA azide through the click reaction (20 mm CuSO_4_, 10 μm TAMRA azide, 10 mm aminoguanidine, and 10 mm ascorbate for 1 h). The resulting TAMRA‐triazole‐labeled proteins, stained with an anti‐TAMRA antibody (1 : 1000, #MA1‐041; Invitrogen), were visualized using either immunoblotting or a confocal microscope. Total actin protein levels were used as a loading control and were detected on the same PVDF membrane. For nascent MYCN detection, the total MYCN protein from l‐HPG treated cells was immunoprecipitated using anti‐MYCN antibodies (as described in the immunoprecipitation). Following this, l‐HPG‐tagged MYCN was captured via the click reaction and subsequently analyzed using immunoblotting. The total immunoprecipitated MYCN was detected and used for normalizing the l‐HPG‐tagged MYCN. Signals from nascent proteins or MYCN were removed before detecting actin or the total immunoprecipitated MYCN on the same membrane. Tumor cells and lysates from l‐HPG‐free cultures were used as negative controls.

### 2.14 Immunoprecipitation

Cells were treated with 25 μm MG132 (Millipore) in methionine‐free RPMI‐1640 supplemented with 200 μm l‐HPG for 2 h and lysed using immunoprecipitation lysis buffer (25 mm Tris–HCl pH 7.4, 150 mm NaCl, 1% NP‐40, 1 mm EDTA, and 5% glycerol) containing protease and phosphatase inhibitors. The protein was collected and incubated with MYCN antibody (1 : 400, #13987; Cell Signaling Technology) or rabbit IgG as the isotype control at 0.5 mg·mL^−1^ in 1 mL at 4 °C for 1 h, with rotation. Subsequently, 20 μL of protein A/G beads (Santa Cruz) were added, and the mixture was further incubated overnight at 4 °C with rotation. The protein A/G beads with immunoprecipitated proteins were collected and washed four times with the washing buffer (0.1% NP‐40 in PBS with 0.02 mm PMSF). The immunoprecipitated proteins were eluded using Lammli buffer, boiled, and subsequently analyzed by western blotting.

### 2.15 Molecular modeling

Protein structures were retrieved from Protein Data Bank (PDB) or Uniprot, with AlphaFold predictions, and were prepared using the Protein Preparation Wizard in Schrodinger suite software. The ligand structures were retrieved from the PDB. In the Schrödinger suite (Schrödinger, Inc, New York, NY, USA), ligprep and epik were used for optimizing ligand structures, and glide v9.1 (using OPLS4 forcefield in XP mode) was used for molecular docking and induced‐fit docking. For proteins with an unknown active site, five docking sites were identified; one to three sites were selected based on Sitescore, Dscore, volume, and balance using the Sitemap tool. The induced‐fit docking was performed using a standard protocol; the MM‐GBSA method was used to compute the binding free energy and the interaction free energy contribution of individual residues to each ceftriaxone fragment (per‐residue energy decomposition). 3D structures were visualized using pymol, and 2D interactions were generated through the Ligand Interaction Diagram in maestro v12.8 (Schrödinger, Inc).

### 2.16 Transcriptomic analysis

RNA extraction and the measurement of RNA quality and quantity were conducted as previously described [17]. RNA libraries were prepared using the TruSeq Stranded mRNA LT Sample Prep Kit (Illumina Inc., San Diego, CA, USA). The RNA sequencing was conducted using the Illumina NovaSeq sequencing system, generating an average of 40 million reads per sample with 100‐bp paired‐end reads. For the processing of RNA‐Seq data, low‐quality reads were removed using trimmomatic (v0.36) (http://www.usadellab.org/cms/?page=trimmomatic). Quantification of the transcriptome was carried out using kallisto (v0.43) (https://pachterlab.github.io/kallisto/) with annotation from Ensembl version 84 at the transcript level. Gene level counts were generated using the tximport r package, and gene count level data were normalized using the TMM (trimmed mean of *M*‐values) method, followed by CPM (counts per million) computation. Differential expression analysis was performed using the exactTest function within the edger r package. Differentially expressed genes were selected based on a log_2_ fold‐change value ≥ 1 and adjusted *P*‐value ≤ 0.01. Subsequently, these genes were subjected to gene set enrichment analysis (GSEA) and GO annotation, using GSEA function with curated gene sets (C2) as Molecular Signatures Database and enrichGO and simplify functions in the clusterprofiler r package (v4.0).

### 2.17 Statistical analysis

IC_50_ and *E*_max_ were evaluated using a two‐sample *t*‐test and Wilcoxon rank sum test, respectively. Colony formation‐based IC_50_ was analyzed using an extra sum‐of‐squares *F*‐test. Multiple groups were compared using a one‐way analysis of variance followed by Dunnett's test (mRNA and protein expression, translation, cell‐cycle, drug toxicity, cell death, and Ki67‐positive cell analyses) or Tukey's test (nascent proteins between tumor cells). Statistical significance was measured at *P* < 0.05.

### 2.18 Study approval

The study methodologies conformed to the standards set by the Declaration of Helsinki and were approved by the Institutional Review Board at the Faculty of Medicine Ramathibodi Hospital, Mahidol University (COA. MURA2015/707 and COA. No. MURA2018/1005). The experiments were undertaken with the understanding and written consent of each subject.

## 3 Results

### 3.1 Ceftriaxone inhibits the growth of *MYCN*‐amplified RB and NB

**Fig. 1:**
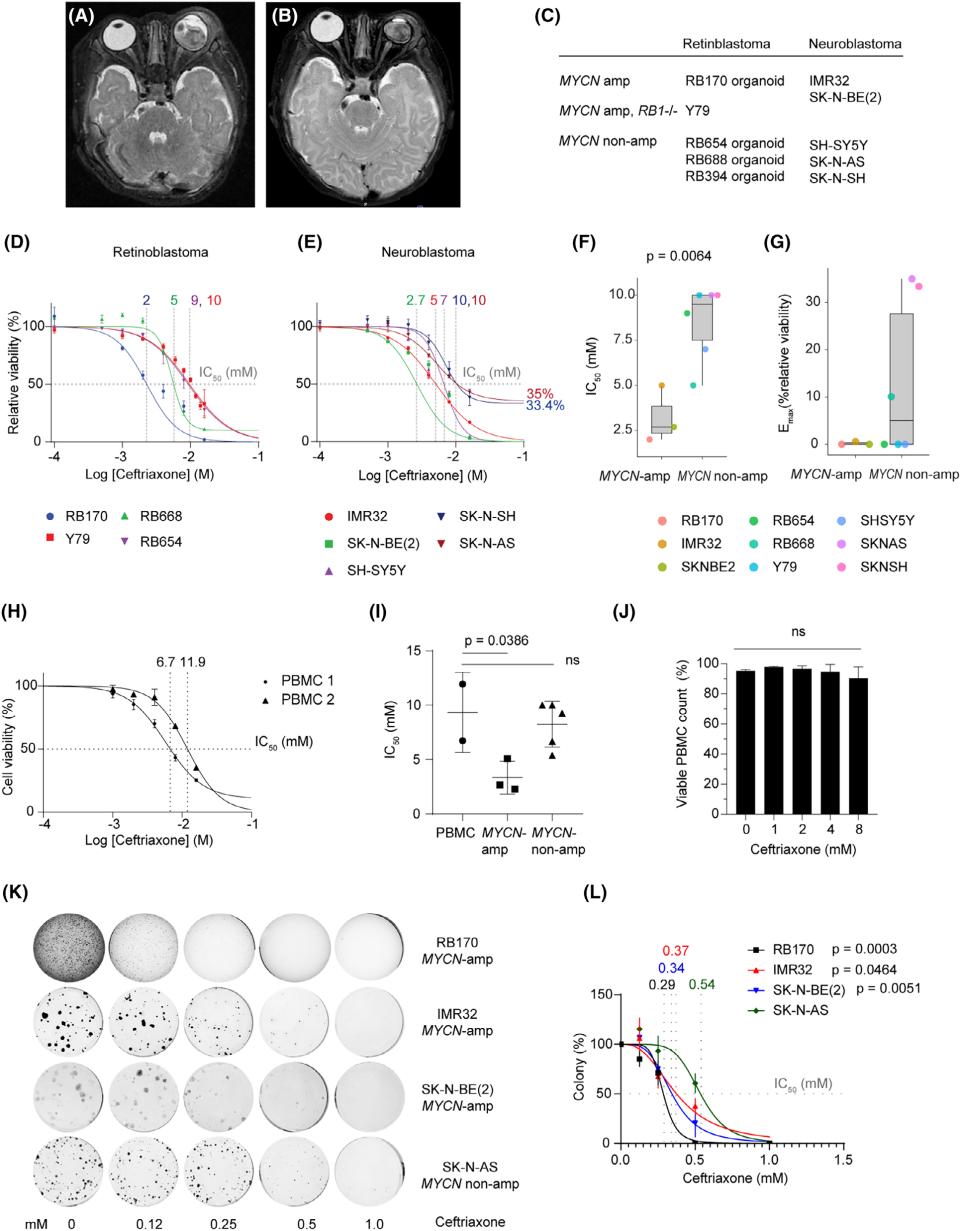
Tumor cells with *MYCN* amplification are susceptible to growth inhibition by ceftriaxone treatment. (A, B) Axial T2‐weighted magnetic resonance imaging shows swelling of left eye tissue on Day 3 (A) and subsequent reduction in ocular mass size on Day 15 (B) postceftriaxone treatment. (C) Retinoblastoma (RB) organoids and neuroblastoma (NB)/RB cell lines used in this study. (D, E) Dose–response curves displaying IC_50_ values (indicated above the graph) for ceftriaxone in RB (D) and NB (E) cells. (F, G) Box plots depicting IC_50_ (F) and *E*_max_ (G) dose–response metrics obtained from D and E. Data were obtained from three independent experiments and analyzed using a two‐sample *t*‐test (IC_50_) and Wilcoxon rank sum test (*E*_max_). (H–J) Drug toxicity assessment in peripheral mononuclear blood cells (PMBCs) from two donors (H), comparing IC_50_ values (indicated above the graph) with those of cancer cells (I), along with viable cell counts for PMBCs postdrug treatment (J). Data (I, J) were obtained from three independent experiments and analyzed using a one‐way analysis of variance followed by Dunnett's test. (K, L) Colony formation assays (K) and dose–response curves (L), including their respective IC_50_ values (indicated above the graph) obtained from colony counts across all concentrations from three independent experiments, were subjected to comparative analysis to identify differences in IC_50_ values between *MYCN*‐amplified and *MYCN*‐nonamplified cells, using the extra sum‐of‐squares *F*‐test. The colony formation data (K) represent three independent experiments. Cells were exposed to ceftriaxone for 48 h (D, E, H–J). Data are shown as mean ± SD *n* = 3 (D–J, L).

We found that intravenously administered ceftriaxone could decrease the size of swollen eyelids/tissue around the left eye initially diagnosed with orbital cellulitis in the patient (RB170) (Fig. 1A,B). However, due to phthisis bulbi, the globe was enucleated and diagnosed as having the MYCN‐driven RB (75 *MYCN* copies) [17]. The patient then received chemotherapy, during which orbital RB was detected, exenterated, and used for generating tumor organoids (RB170 organoids), as reported previously [17].

Based on the clinical observations, we examined the antitumor activities of ceftriaxone in *MYCN*‐amplified RB170 organoids compared with those in *MYCN‐*nonamplified RB organoids (RB668 and RB654) [17, 18]. The Y79, the classical RB subtype carrying *MYCN* amplification, was also used for this examination (Fig. 1C). Ceftriaxone exhibited high potency against RB170, as indicated by a low IC_50_ value compared with the IC_50_ values in the other RB cells (Fig. 1D). These data suggest that MYCN‐driven RB is more susceptible to ceftriaxone‐induced growth suppression than the *RB1*‐deficient RB with or without *MYCN* amplification.

Similarly, *MYCN*‐amplified NB cells [IMR32 and SK‐N‐BE (2)] were more vulnerable to growth inhibition by antibiotic treatment than *MYCN*‐nonamplified NB cells (SH‐SY5Y, SK‐N‐AS, and SK‐N‐SH), as indicated by the lower IC_50_ values (Fig. 1C,E). Given the lower IC_50_ and *E*_max_ values, ceftriaxone had high potency and efficacy, respectively, in *MYCN*‐amplified tumor cells compared with that in *MYCN‐*nonamplified tumor cells (Fig. 1F,G). Drug potency also indicated that the response of MYCN‐driven RB to ceftriaxone was distinguishable from other RB tumor cells but similar to NB with *MYCN* amplification (Fig. 1F).

A drug toxicity test in healthy PBMCs revealed lower sensitivity to ceftriaxone compared with *MYCN*‐amplified cancer cells but similar sensitivity to *MYCN*‐nonamplified cancer cells, as indicated by the IC_50_ values (Fig. 1H,I). Furthermore, the number of viable cells in drug‐treated PBMC cultures remained consistent with that in vehicle‐treated PBMC cultures, even at elevated drug concentrations (Fig. 1J). This underscores the favorable toxicity profile of ceftriaxone in normal cells.

We then examined the effects of the drug using doses based on plasma concentrations (0.2–0.5 mm) of ceftriaxone in infants aged 1–24 months, with dosages ranging from 210 to 950 mg per dose [20], and treatment durations typically used for patients (14 days or longer) in colony formation assays. Consistent with the viability‐based IC_50_ values, the dose of ceftriaxone used to inhibit 50% colony formation of *MYCN*‐amplified tumor cells was lower than that used in *MYCN‐*nonamplified tumor cells, confirming growth inhibition of *MYCN*‐driven tumors upon ceftriaxone treatment (Fig. 1K,L). Notably, ceftriaxone doses (IC_50_ of 0.29 –0.37 mm) and treatment length corresponded to clinically achievable doses and duration of antibiotic therapy in infants [20], implying that the plasma concentrations are potent enough to inhibit tumor growth as observed in the RB170 patient with age at diagnosis of 3 months (Fig. 1A,B,K,L). The IC_50_ values from each assay consistently indicated increased drug sensitivity in *MYCN*‐driven tumor cells and were dependent on the duration of antibiotic treatment. Notably, the IC_50_ values from the assays with a 48‐h drug treatment were higher than those from the 2‐week prolonged drug treatment (Fig. 1D,E,L). Given the treatment length, the viability‐based IC_50_ from the ATP assays with shorter drug treatment was used to determine drug concentrations for subsequent experiments.

### 3.2 Cell‐cycle progression and survival are suppressed during ceftriaxone treatment

Transcriptomic analysis was conducted to evaluate the effect of ceftriaxone in *MYCN*‐amplified tumor cells. Overall, 467 (268 upregulated and 199 downregulated genes) and 2063 (757 upregulated and 1306 downregulated genes) differentially expressed genes (DEGs) were identified from RB170 organoids treated with 2‐mm (IC_50_) and 8‐mm (4× IC_50_) ceftriaxone, respectively, compared with vehicle‐treated cells. Notably, *NGFR* linked to spontaneous regression in RB and NB [21, 22] was the most upregulated gene with a 56‐ (IC_50_) and 85‐ (4× IC_50_) fold change following antibiotic treatment (Fig. S1A).

**Fig. 2:**
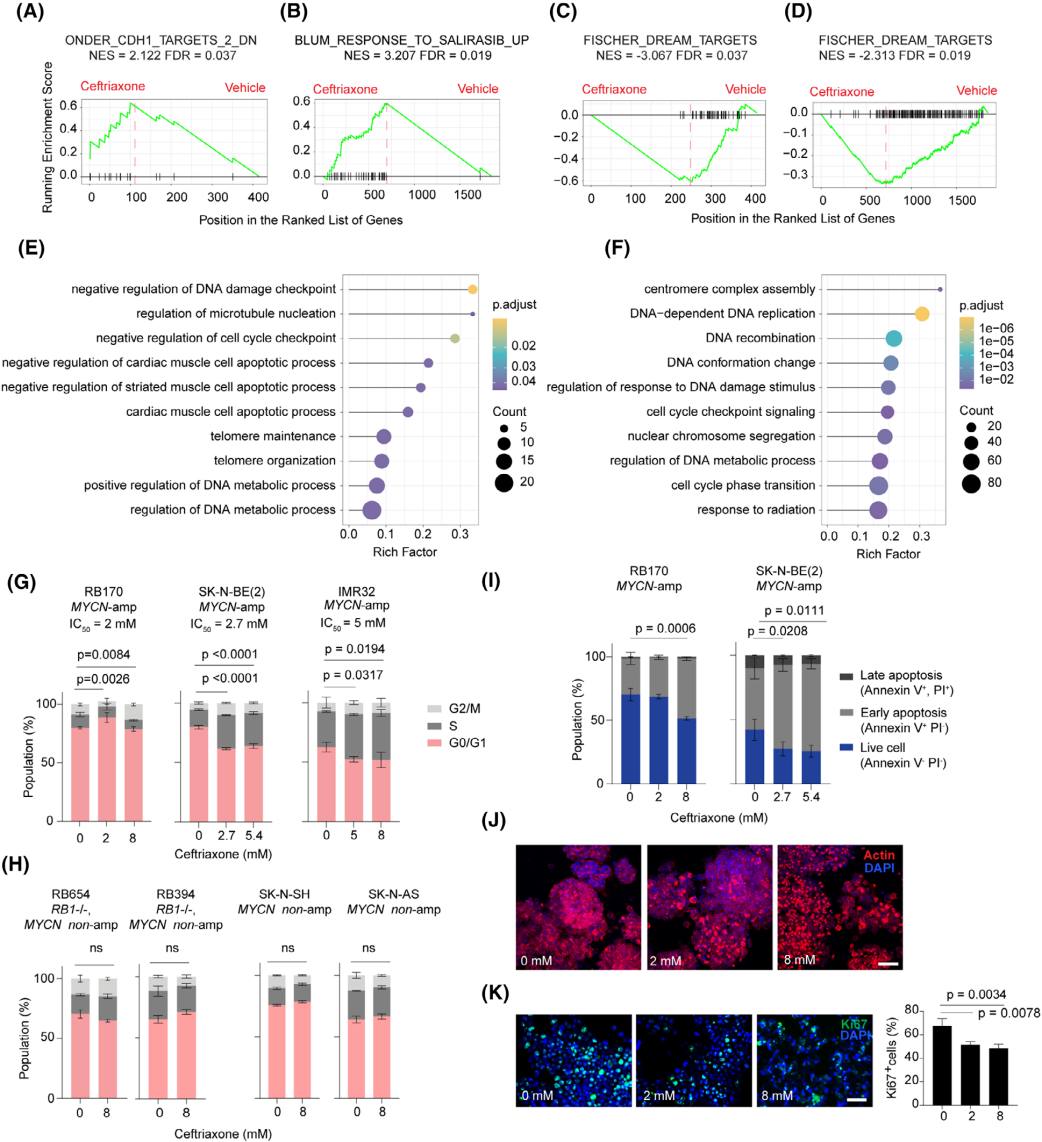
Gene signature, cell‐cycle, and death profiles indicate anticancer activities of ceftriaxone in *MYCN*‐amplified tumor cells. (A–D) Gene set enrichment analysis (GSEA) showed the most significantly enriched gene sets positively (A, B) and negatively (C, D) correlated with a gene set from RB170 treated with 2 (A, C) or 8 (B, D) mm drug. NES, normalized enrichment score. Data were derived from two independent experiments. (E, F) Gene Ontology analysis enriched the top 10 terms from RB170 treated with 2 (E) or 8 (F) mm ceftriaxone. (G, H) Cell‐cycle profiles for *MYCN*‐amplified (G) and *MYCN*‐nonamplified (H) cells. (I) Apoptotic cell death profiles for *MYCN*‐amplified cells. (J, K) Whole‐mount actin staining (J) and Ki67 staining (K) of RB170 organoids with and without drug treatment. Analysis of Ki67‐positive cells involved counting approximately 2400–5000 cells from 9 to 12 microscope fields (three to four fields/experiment from three independent experiments) for each treatment. Scale bar: 50 μm (J, K). For all experiments, cells were exposed to ceftriaxone for 48 h. Data (G–I, K) were obtained from three independent experiments and analyzed using a one‐way analysis of variance, followed by Dunnett's test. Data are shown as mean ± SD, *n* = 3 (G–I, K).

Gene set enrichment analysis revealed that a gene set of ONDER_CDH1_TARGETS_2_DN and BLUM_RESPONSE_TO_SALIRASIB_UP had the highest normalized enrichment score (NES) and positively correlated with a gene set from tumor cells with 2‐ and 8‐mm ceftriaxone treatment, respectively (Fig. 2A,B; Fig. S1B,C). The results suggest that ceftriaxone can upregulate the expression of genes associated with the function of CDH1, which encodes E‐cadherin—an important factor known for maintaining epithelial phenotypes and contributing to antimetastasis. Furthermore, the upregulated genes are associated with cancer cell response to salirasib, a Ras inhibitor with antitumor activities [23]. Interestingly, the FISCHER_DREAM_TARGETS gene set had the lowest NES and inversely correlated with the gene expression from tumor cells with ceftriaxone treatment at both doses (Fig. 2C,D). This result suggests that ceftriaxone might have the potential to downregulate the expression levels of DREAM‐target genes, which include G1/S and G2/M cell‐cycle genes [24], potentially affecting cell‐cycle progression.

Gene Ontology analysis showed that ‘negative regulation of DNA damage checkpoint’ and ‘DNA repair and cell replication’ were the most significantly overrepresented terms and were associated with downregulated genes after 2‐ and 8‐mm ceftriaxone treatment, respectively (Fig. 2E,F; Fig. S1D,E). Additionally, the terms enriched among the downregulated genes were indicative of cell‐cycle inhibition and apoptosis (Fig. 2E,F; Fig. S1D,E). These GO terms collectively supported the observed downregulation of cell‐cycle genes in GSEA (Fig. 2C,D). Furthermore, the upregulated genes showed enrichment for ‘positive regulation of transcription from RNA polymerase II promoter in response to (endoplasmic reticulum) stress’ as the most significantly enriched terms (Fig. S1F). Altogether, transcriptomic analysis suggests that ceftriaxone treatment induces cellular stress and inhibits cell‐cycle progression, leading to growth suppression and death of *MYCN*‐amplified tumors.

We performed cell‐cycle and apoptotic analysis assays to confirm our findings from the transcriptomic analysis. We detected cell‐cycle arrest at G0/G1 (IC_50_) and G2/M (4× IC_50_) after ceftriaxone treatment of RB170 (Fig. 2G). S‐phase arrest was detected in drug‐treated SK‐N‐BE (2) and IMR32 cells (Fig. 2G). Intriguingly, cell‐cycle progression remained unaffected in *MYCN*‐nonamplified RB and NB cells when exposed to the highest dose of the drug (8 mm) applied to *MYCN*‐driven tumor cells (Fig. 2H). These findings provided further supporting evidence for the drug's specificity toward *MYCN*‐driven tumor cells. Ceftriaxone could induce apoptotic death of RB170 (at 4× IC_50_) and SK‐N‐BE (2) (Fig. 2I). Whole‐mount actin staining revealed RB170 organoid shrinkage, correlating with a reduction in Ki67‐positive proliferative cells (Fig. 2J,K). This decrease in Ki67‐positive cells was also confirmed in SK‐N‐BE (2) (Fig. S1G). The results from the transcriptomic, cell‐cycle, and death profiles and actin and Ki67 staining corroborated the anticancer activities of ceftriaxone in the RB170 patient (Figs 1A,B and 2G–K).

### 3.3 MYCN protein expression is repressed by ceftriaxone treatment

**Fig. 3:**
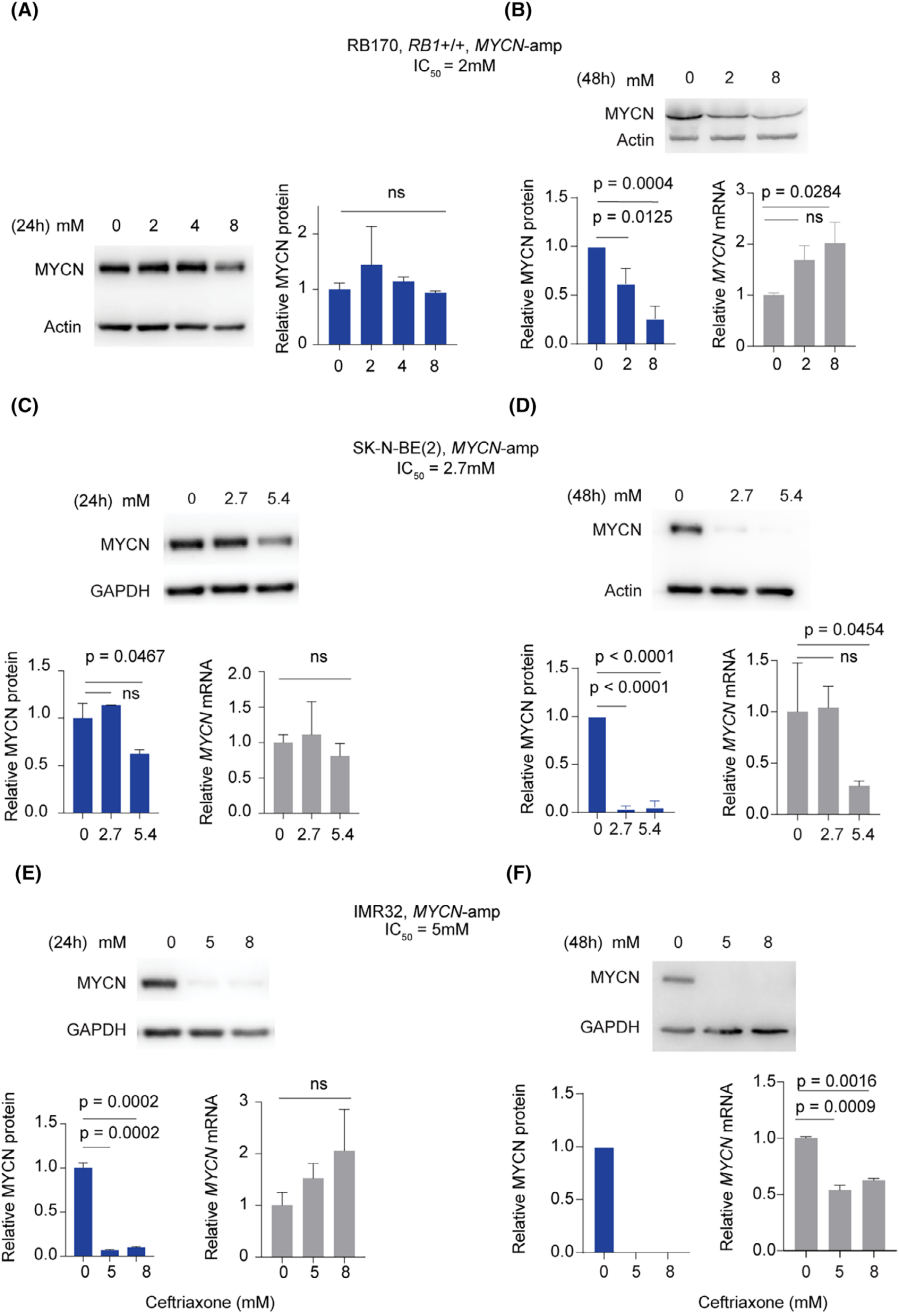
MYCN protein expression is repressed by ceftriaxone treatment. (A–F) Detection of MYCN protein by western blotting and mRNA by RT‐qPCR in RB (A, B) and NB (C–F) cells. Data were obtained from three independent experiments and analyzed using a one‐way analysis of variance, followed by Dunnett's test. All data are shown as mean ± SD, *n* = 3.

We analyzed MYCN protein expression in cancer cells to investigate the influence of *MYCN* status on drug response. Our findings reveal that *MYCN*‐amplified RB and NB cells exhibited MYCN overexpression (Fig. S2A). In contrast, *MYCN*‐nonamplified RB and NB cells either lacked detectable MYCN expression or had low MYCN expression compared with their *MYCN*‐amplified counterparts. This suggests that the correlation between a higher number of *MYCN* gene copies and increased sensitivity to ceftriaxone can be attributed to MYCN protein overexpression (Figs 1F,L and 2G,H; Fig. S2A). We then examined whether ceftriaxone could affect MYCN in RB and NB after treatment for 24 or 48 h. After 48 h of ceftriaxone treatment, MYCN protein levels in RB170 decreased, in contrast to the 24‐h treatment (Fig. 3A,B). However, *MYCN* mRNA levels remained unaltered at the IC_50_ but increased at 4× IC_50_ after 48‐h treatment (Fig. 3B). These findings suggest that ceftriaxone induces post‐transcriptional downregulation of MYCN. In SK‐N‐BE (2), MYCN protein levels were significantly reduced after antibiotic treatment for 24 h (2× IC_50_) and 48 h (1× and 2× IC_50_), but the mRNA levels remained unaffected except for the 48‐h treatment at 2× IC_50_ (Fig. 3C,D). In IMR32, MYCN protein was virtually absent after treatment for 24 h and was repressed with prolonged exposure for 48 h (Fig. 3E,F). *MYCN* transcripts remained unaffected after treatment for 24 h but were significantly reduced after the 48‐h treatment in IMR32 (Fig. 3E,F). The reduction in *MYCN* mRNA levels in SK‐N‐BE (2) and IMR32 (Fig. 3D,F) could be related to the inhibition of positive autoregulation of *MYCN* gene following complete repression of MYCN protein at 48‐h treatment [25]. Altogether, ceftriaxone can post‐transcriptionally downregulate MYCN expression levels in *MYCN*‐amplified tumor cells.

Given that *MYCN*‐nonamplified NB cells express c‐MYC (Fig. S2A), we examined the impact of ceftriaxone on c‐MYC levels and its correlation with drug sensitivity. When *MYCN*‐nonamplified NB cells were exposed to a 2 mm dose of ceftriaxone, which effectively suppressed MYCN in *MYCN*‐amplified NB cells, the levels of c‐MYC remained unchanged in these cells (Fig. S2B,C). This lack of c‐MYC suppression corresponded with higher IC_50_ values, indicating reduced drug sensitivity (Fig. 1F,L). The reduced drug sensitivity observed in *MYCN*‐nonamplified NB cells, despite the presence of c‐MYC but the absence of MYCN, suggests the critical role of MYCN in mediating drug effects.

### 3.4 Drug–target interaction network analysis reveals target proteins of ceftriaxone in *MYCN*‐amplified tumor cells

**Fig. 4:**
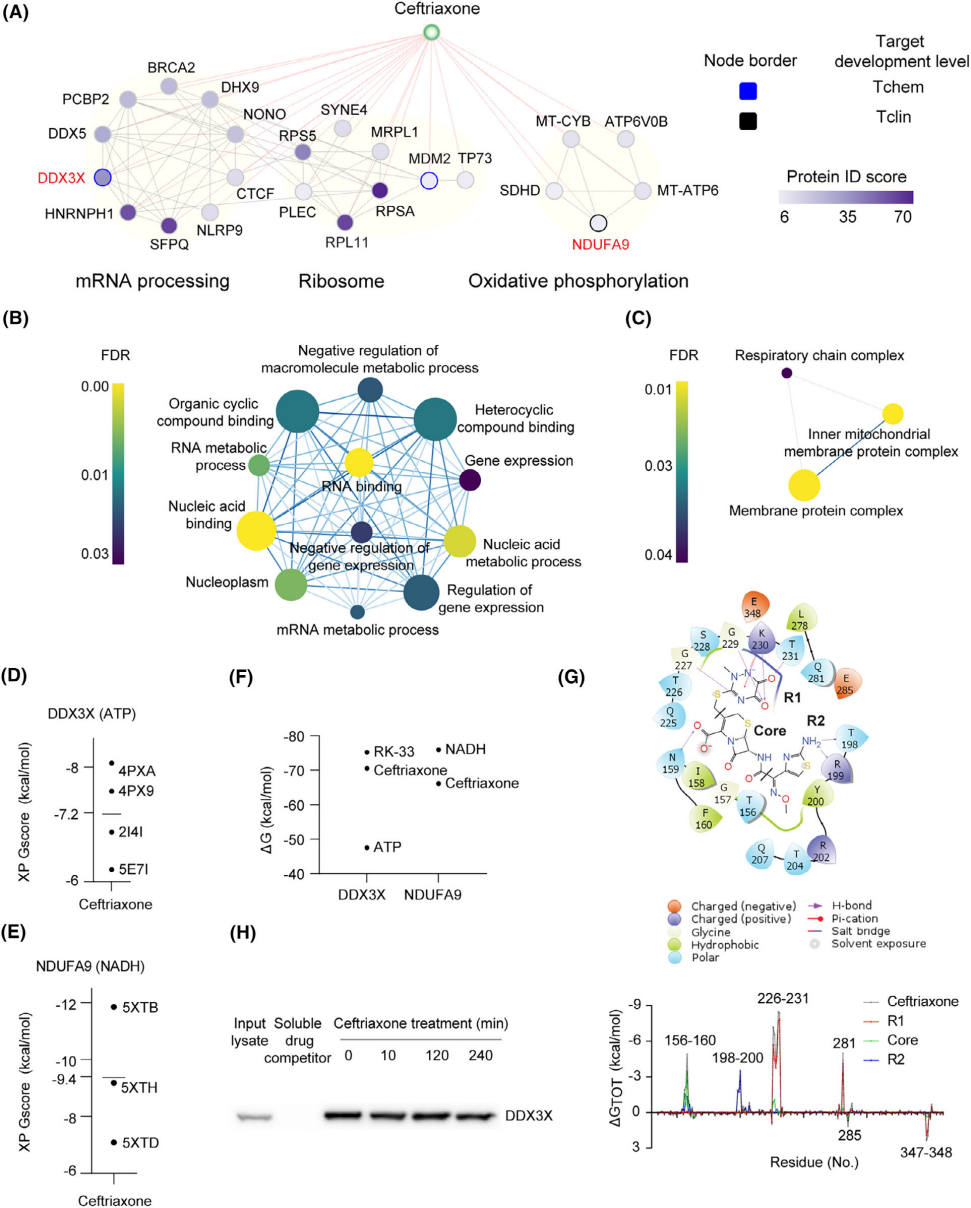
DDX3X is identified as the protein target of ceftriaxone. (A) Drug–target interaction (DTI) networks with the most significantly enriched pathway. A higher protein ID score indicates a more confident match of amino acid sequences within the target protein. Tchem is a protein known to bind small molecules with high potency; Tclin is a protein via which approved drugs act. (B, C) Gene Ontology analysis of DTI networks. A darker blue node‐linking edge indicates more genes overlapping between the two nodes. The size of the node represents the gene size. (D, E) Cross‐docking of ceftriaxone toward DDX3X (D) at the ATP‐binding cleft of DDX3X and NDUFA9 (E) at the NADH‐binding cleft of NDUFA9. PDB IDs are shown, referring to the identifier of protein structures, and the black line represents the mean docking score. (F) Induced‐fit docking of ceftriaxone and other ligands toward DDX3X and NDUFA9. (G) A predicted molecular model of ceftriaxone docked into the ATP‐binding cleft of DDX3X. The 2D ligand interaction diagram was generated using the Ligand Interaction Diagram in maestro v12.8. Per‐residue energy decomposition was used to predict the contribution of each residue of DDX3X to ceftriaxone interaction. (H) DDX3X detection by western blot analysis of drug‐binding proteins from the lysates of RB170. A protein sample with a soluble drug competitor (200 mm) is defined as a nonspecific drug‐binding protein. These data represent two independent experiments.

Drug‐binding proteins were identified and used for generating a DTI network to illustrate a mechanism of action for ceftriaxone. *In situ* drug action was initiated by ceftriaxone treatment of RB170 tumor organoids. Drug‐binding proteins were isolated from lysates using drug‐conjugated beads and were subjected to proteomic analysis. The proteins that bound drug‐conjugated beads in the presence of soluble drug competitors were excluded as nonspecific drug‐binding proteins. With the confidence (protein ID score) cutoff set to > 6 (mean ID), a total of 171, 144, 133, 131, 164, 163, and 144 proteins was identified after ceftriaxone treatment for 10 min, 2, 4, 8,16, 24, and 48 h, respectively (Table S1), and was analyzed to generate DTI networks. We found that the DTI networks were consistent from each time point of antibiotic treatment (Fig. S3A–G). Merging the networks from each condition to generate a core network resulted in three DTI networks, two of which were connected (Fig. 4A). Functional analysis of the DTI networks demonstrated enrichment in the pathways related to mRNA processing, ribosome, and oxidative phosphorylation (OXPHOS) (Fig. 4A).

Concordantly, GO analysis showed enrichment in ‘RNA binding’ for the DTI networks associated with mRNA processing and ribosomes and ‘inner mitochondrial membrane protein complex’ for the OXPHOS network as the most significantly enriched term (Fig. 4A–C). Additionally, the ‘nucleic acid/RNA/mRNA metabolic process’ was overrepresented within a set of proteins from the DTI networks (Fig. 4A,B). Remarkably, the identified proteins, indicative of mRNA metabolism, included RNA helicases (DDX3X, DHX9, and DDX5) that can bind and unwind the structured 5′ untranslated regions (UTRs) in the mRNAs to increase ribosomal occupancy of the mRNAs during translation (Fig. 4A) [26]. Other proteins included SFPQ, which modulates 5′‐UTR mediated translational control [27], and ribosomal proteins (RPL11, RPSA, RPS5, and MRPL1), which can bind the ribosome assembly factor RNA helicases [28, 29]. Several mitochondrial proteins (NDUFA9, MT‐CYB, MT‐ATP6, SDHD, and ATP6V0B) were observed in the DTI networks. Collectively, data suggest that translation control and mitochondrial function are relevant to the mechanisms of anticancer action for ceftriaxone.

### 3.5 Structure‐based visual screening of drug‐binding proteins identifies DDX3X as a molecular target of ceftriaxone

Next, we performed molecular docking to predict interactions of ceftriaxone with the proteins identified in the DTI networks (Fig. 4A). Compared with other proteins, ceftriaxone exhibited high binding affinity toward DDX3X and NDUFA9, which was confirmed by cross‐docking with multiple protein structures of both proteins (Fig. 4D,E; Fig. S4A and Table S2). DDX3X and NDUFA9 were predicted to have stronger interactions with ceftriaxone than AURKA and AURKB, which were shown to bind ceftriaxone in a previous study [16] (Fig. S4A–C).

We further predicted the binding affinities of ceftriaxone in comparisons with endogenous ligands (NADH and ATP) and RK‐33, a small molecule inhibitor binding to the ATP‐binding cleft of DDX3X [30], toward NDUFA9 and DDX3X by induced‐fit docking. Given the high free energy of ceftriaxone compared with NADH, the drug might not be able to compete with the endogenous ligand for binding at the NADH‐binding cleft of NDUF9 and is thus incapable of functional inhibition of NDUFA9 (Fig. 4F). Additionally, a mitochondrial ATP detection assay was conducted in RB170 and SK‐N‐BE(2) cells to confirm that ceftriaxone does not interfere with NADH binding in NDUFA9 within the electron transport chain (Fig. S5A,B). Interestingly, ceftriaxone and RK‐33 had similar binding affinities for DDX3X and had lower free energy than ATP (Fig. 4F). These results suggest that ceftriaxone akin to RK‐33 potentially competes with ATP for binding at the ATP‐binding cleft and can thereby inhibit the function of ATP‐dependent DDX3X helicase [30] (Fig. 4G). The R2 moiety of ceftriaxone provided the highest stabilization for DDX3X binding (at residues 226–231 and 281) (Fig. 4G), arguing for ceftriaxone's higher potency and efficacy when compared to cefotaxime and cefepime, which have exact structures except for R2 (Fig. S5C,D). Ceftriaxone was also superior to cefazolin and ceftazidime, a first‐ and third‐generation cephalosporin, in *MYCN*‐amplified RB170 organoids (Fig. S5D). These results suggest that the observed anticancer effect is specific to ceftriaxone binding to DDX3X.

Ceftriaxone‐DDX3X binding was confirmed in RB170 by western blotting; no DDX3X band was detected for the reaction with the drug competitor, indicative of specific drug‐protein interaction (Fig. 4H). Identification of DDX3X as the drug target suggests that translation control rather than mitochondrial function was associated with the mechanism of anticancer action for ceftriaxone.

### 3.6 High DDX3X expression levels are associated with poor prognosis

**Fig. 5:**
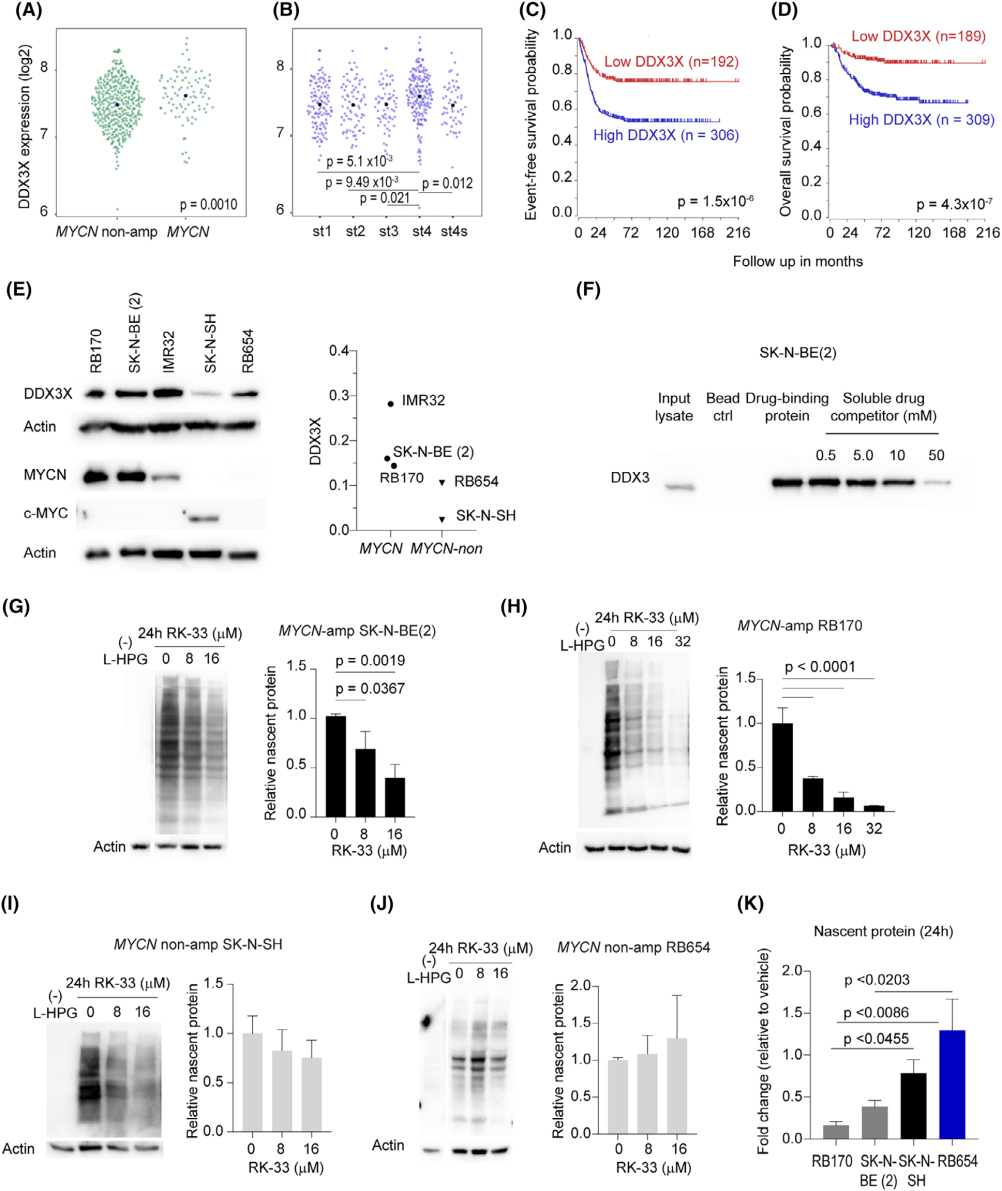
High *DDX3X* expression is associated with poor prognosis, and DDX3X inhibition represses translation in *MYCN*‐amplified tumors. (A–D) High *DDX3X* expression levels in neuroblastoma (NB) were associated with *MYCN* amplification (A), advanced INSS stages (B), and poor event‐free (C)/overall (D) survival. Each point on the plots (A, B) represents *DDX3X* levels in individual NB, and mean values are shown. *DDX3X* mRNA expression cutoff was computed by Kaplan scanner to separate patient survival into two groups; the log‐rank test was used for survival analysis (C, D). The analysis was performed on publicly available data (Tumor Neuroblastoma SEQC‐498‐RNA‐Seq) from R2: Genomic Analysis and Visualization Platform (http://r2.amc.nl). (E) Representative western blots show protein expression profiles from tumor cells with and without *MYCN* amplification. These data represent two independent experiments. (F) DDX3X detection by western blot analysis of drug‐binding proteins from the lysates of NB cells. A soluble drug competitor was used to test a specific drug–protein interaction; a protein sample eluted from beads without drug conjugation (bead ctrl) is a negative control. These data represent two independent experiments. (G–J) Translation assays show l‐HPG‐labeled proteins after RK‐33 treatment for 24 h of *MYCN*‐amplified NB (G) and retinoblastoma (RB) (H) and *MYCN*‐nonamplified NB (I) and RB (J). A sample without l‐HPG incorporation into the nascent proteins is a negative control. Data were obtained from three independent experiments and analyzed using a one‐way analysis of variance followed by Tukey's test. (K) Comparisons were made for nascent proteins obtained from G to J (16 μm RK‐33). The data were subjected to analysis using a one‐way analysis of variance, followed by Tukey's test. Data are shown as mean ± SD, *n* = 3 (G–K). The lanes (gaps in G, J) were run on the same gel but were noncontiguous.

Next, we analyzed a large RNA seq dataset (SEQC‐498) from 498 NB tumors to examine the association of *DDX3X* with tumor aggression. High *DDX3X* expression levels were associated with *MYCN* amplification and advanced‐stage disease (INSS 4) (Fig. 5A,B). Kaplan–Meier analyses showed that patients with high *DDX3X* expression levels in NB had poor event‐free and overall survival (Fig. 5C,D). These results were consistent with other independent NB cohorts investigated (Fig. S6). However, due to limitations in the available data from existing RB studies, we are unable to establish the association of *DDX3X* in RB tissue with survival rates. In our study, protein expression profiling showed that *MYCN*‐amplified NB and RB with overexpressed MYCN had higher DDX3X levels compared with their nonamplified counterparts (Fig. 5E). These data suggest that DDX3X is associated with *MYCN* amplification, the strongest predictor of high‐risk NB, and might be a novel target for treating *MYCN*‐driven tumors.

### 3.7 Inhibition of DDX3X represses translation in *MYCN*‐amplified tumor cells

As observed with RB170, we found that ceftriaxone interacted with DDX3X in *MYCN*‐amplified NB; DDX3X levels decreased as the concentration of a soluble drug competitor increased, indicative of a specific ceftriaxone‐DDX3X interaction (Fig. 5F). Given the DDX3X identified as the drug target, we hypothesized that the translation was affected by the functional inhibition of DDX3X (Fig. 4F,G). To prove the hypothesis, an activity of DDX3X was inhibited with small molecule inhibitor RK‐33, and translation was then examined through l‐HPG incorporation into the nascent proteins (Fig. S7A). We found that l‐HPG incorporation was significantly inhibited, indicating the blocking of protein synthesis in *MYCN*‐amplified SK‐N‐BE (2) and RB170 after 24‐h treatment with RK‐33 (Fig. 5G,H). These results corroborated target identification.

Interestingly, the translation in *MYCN*‐nonamplified SK‐N‐SH and RB654 was not significantly suppressed (Fig. 5I,J). These results indicated that *MYCN*‐amplified RB and NB cells were more susceptible to translation repression by RK‐33‐mediated DDX3X inhibition than *MYCN*‐nonamplified tumor cells (Fig. 5K). The MYCN‐status‐dependent sensitivity of tumor cells to RK‐33 (Fig. 5E,K) suggests selective effects of DDX3X inhibition on *MYCN*‐amplified tumors.

### 3.8 The translation is repressed by DDX3X‐targeting ceftriaxone in *MYCN*‐amplified RB and NB

**Fig. 6:**
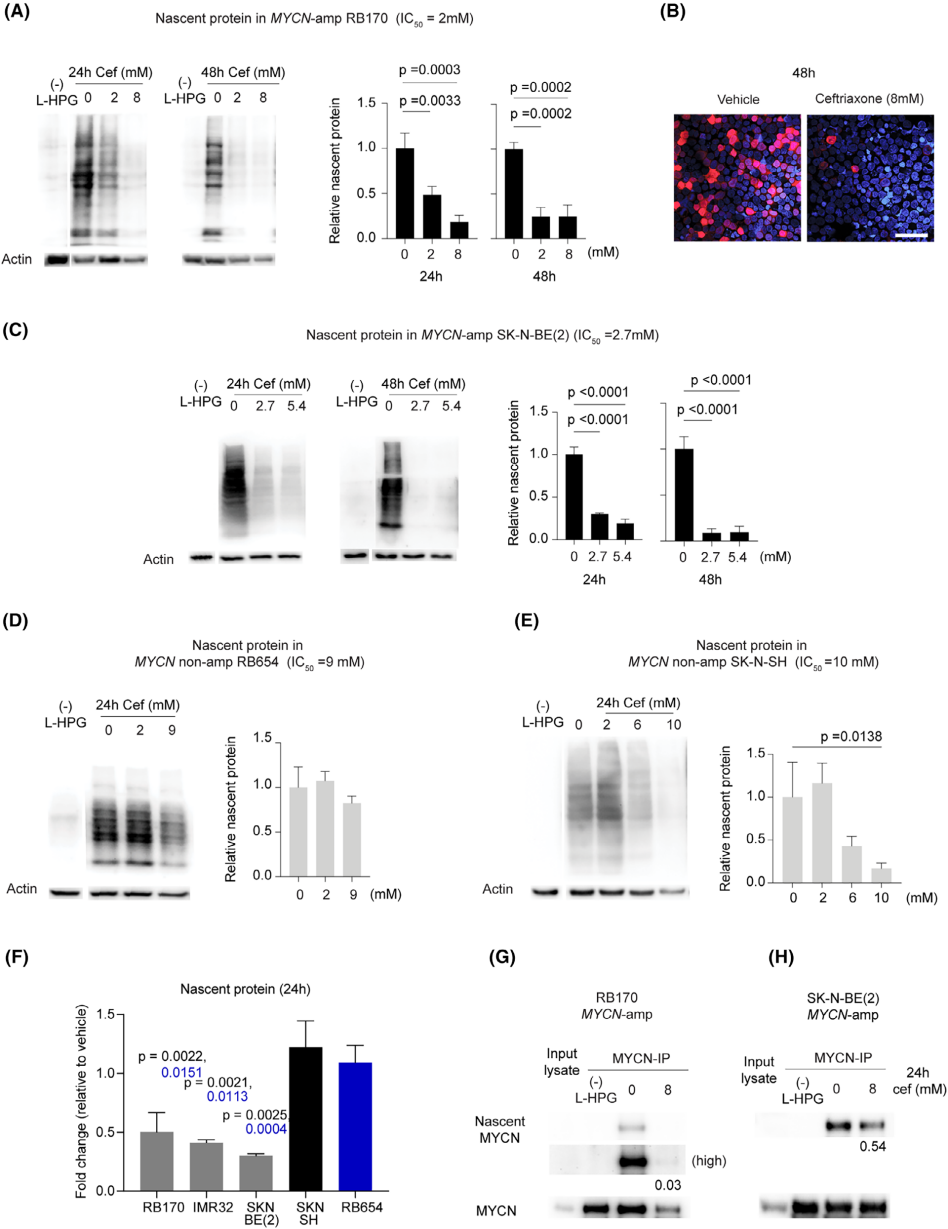
The translation is repressed by DDX3X‐targeting ceftriaxone in *MYCN*‐amplified retinoblastoma (RB) and neuroblastoma (NB). (A–E) Translation assays demonstrate the presence of l‐HPG‐labeled proteins in *MYCN*‐amplified tumors RB170 (A, B) and SK‐N‐BE (2) (C), as well as in *MYCN*‐nonamplified tumors RB654 (D) and SK‐N‐SH (E). The detection of l‐HPG‐labeled proteins was achieved using both immunoblotting (A, C–E) and immunofluorescence. (B). Cef, ceftriaxone. A sample without l‐HPG incorporation into the nascent proteins is a negative control. Scale bar: 50 μm (B). Data (A, C–E) were obtained from three independent experiments and analyzed using a one‐way analysis of variance, followed by Tukey's test. The immunofluorescence data (B) represent two independent experiments. (F) Comparisons were made for nascent proteins obtained from A, C–E, and Fig. S7B (about 2‐mm ceftriaxone treatment). The data were subjected to analysis using a one‐way analysis of variance, followed by Tukey's test. (G, H) Representative blots show l‐HPG‐tagged (nascent) MYCN protein from RB170 (G) and SK‐N‐BE (2) (H). The levels of nascent MYCN were normalized to the levels of immunoprecipitated MYCN. Relative nascent MYCN (to no treatment) is shown; (high) in (G) indicates detection under long exposure time; MYCN‐IP: immunoprecipitated MYCN proteins. These data represent two independent experiments. Data are shown as mean ± SD, *n* = 3 (A, C–F). The lanes (gaps in A, C–E) were run on the same gel but were noncontiguous.

Next, we examined whether the ceftriaxone treatment inhibited translation as suggested by target identification and validation. As observed with RK‐33, we found that l‐HPG incorporation was significantly inhibited in *MYCN*‐amplified RB170, SK‐N‐BE (2), and IMR32 after 24‐ and 48‐h treatment with ceftriaxone (Fig. 6A–C; Fig. S7B). The levels of l‐HPG‐tagged proteins were further reduced after prolonged treatment for 48 h (Fig. 6A–C). However, translation in *MYCN*‐nonamplified tumor cells (RB654 and SK‐N‐SH) was not significantly affected by ceftriaxone treatment, even at the high dose (9 mm ceftriaxone) in RB654 (Fig. 6D,E). We found that an increased concentration of ceftriaxone (10 mm) was required to repress translation in SK‐N‐SH cells which express c‐MYC rather than MYCN (Figs 5E and 6E). Thus, DDX3X inhibition by ceftriaxone and RK‐33 demonstrated the same effect on translation.

As observed with RK‐33, *MYCN*‐amplified tumors were most susceptible to translation repression by ceftriaxone, followed by c‐MYC‐expressing tumor cells treated with high drug doses (Figs 5K and 6F; Fig. S7C). We found that MYCN overexpression was associated with the sensitivity of tumor cells to ceftriaxone treatment (Figs 5E and 6F; Fig. S7C). Our evidence indicated that ceftriaxone‐induced translation repression was specific to the *MYCN*‐amplified tumors, providing selective anticancer effects.

Furthermore, we examined l‐HPG‐tagged MYCN from immunoprecipitated MYCN proteins to determine the effect of ceftriaxone on MYCN translation (Fig. S7A). We found that MYCN translation was inhibited after antibiotic treatment for 24 h in RB170 and SK‐N‐BE (2) (Fig. 6G,H). Collectively, ceftriaxone could inhibit translation via targeting DDX3X, and concomitant inhibition of MYCN overexpression by drug treatment resulted in a significant repression of translation in *MYCN*‐amplified tumor cells.

## 4 Discussion

From the clinical observation that ceftriaxone treatment can decrease the volume of unexpected *MYCN*‐driven RB, we demonstrate that ceftriaxone similarly has antitumoral activities in RB and NB cells and is a potent growth inhibitor of *MYCN*‐amplified tumor cells. Ceftriaxone exhibits selectivity in targeting *MYCN*‐amplified tumors. As a mechanism, ceftriaxone binding and inactivating the target protein DDX3X resulted in translation repression associated with concomitant inhibition of MYCN overexpression in *MYCN*‐amplified tumors but not in *MYCN* wild‐type tumors. Our data indicate the feasibility of repurposing ceftriaxone as an anticancer drug and DDX3X as a novel target for treating these tumors.

*MYCN*‐driven RB and NB display the unique molecular signatures associated with mRNA translation and cell‐cycle progression [9], which can be targeted by ceftriaxone treatment, as shown in this study. Inhibition of the elevated rate of translation with a small molecule inhibitor is a promising strategy for suppressing MYCN‐dependent tumor growth and is reportedly effective for controlling the high‐risk *MYCN‐*driven NB [31, 32]. Our study agrees that translation has therapeutic vulnerabilities that can be targeted to inhibit MYCN‐dependent tumor growth and provides a potential anticancer strategy using this FDA‐approved drug as an alternative to a small molecule translation inhibitor for treating *MYCN*‐amplified pediatric cancers.

The MYC oncogene family coordinates protein synthesis in *MYC/N*‐driven cancer cells by monopolizing the production of ribosomal components and controlling protein folding to cope with altered protein synthesis [33, 34, 35]. Thus, inhibition of MYCN overexpression directly affects protein synthesis, explaining more susceptibility to ceftriaxone of *MYCN*‐amplified tumors compared with other tumors without gene amplification/overexpression. We observed a reduced effect of drug treatment on translation blocks and growth inhibition in *MYCN*‐nonamplified NB cells expressing c‐MYC. This is consistent with previous reports on inhibition of translation via suppressing ribosome biogenesis; the growth of c‐MYC‐expressing NB cells can be suppressed to a lesser extent relative to *MYCN*‐amplified NB cells [31, 32]. *c‐MYC*, which has the overall structure and organization of genes and transcripts similar to *MYCN* [36, 37], may be responsible for partial response to ceftriaxone of tumor cells expressing the c‐MYC rather than MYCN. We showed that *MYCN*‐amplified tumor cells had higher levels of DDX3X expression than *MYCN*‐nonamplified tumor cells. This DDX3X overexpression may sensitize tumor cells to ceftriaxone treatment in addition to MYCN overexpression.

Overexpression of DDX3X, here identified as a drug target, has been reported in many cancers and is associated with poor survival rates of cancer patients [30, 38, 39, 40]. Depletion of DDX3X inhibits protein synthesis [38, 41, 42], consistent with the observed translation repression after ceftriaxone and RK‐33 treatment of *MYCN*‐amplified tumor cells in our study. Additionally, DDX3X inactivation with a small molecule inhibitor RK‐33 induces a global delay of cell‐cycle progression during interphase and mitosis and increases cancer cell death [30, 43, 44, 45]. Our findings from the drug‐induced transcriptomic and cell‐cycle and death profiles are consistent with these previous studies and corroborate that DDX3X is the protein target of ceftriaxone. Notably, ceftriaxone akin to RK‐33 had binding affinities for the ATP‐binding cleft of DDX3X. Collectively, ceftriaxone binding DDX3X might be able to inhibit the ATP hydrolysis‐dependent DDX3X function, resulting in impaired translational control and cell‐cycle progression.

In the context of cancer growth, the functional interplay between *MYCN* and *DDX3X* remains enigmatic. MYCN significantly enhances protein synthesis, particularly in *MYCN‐*driven cancers. DDX3X is an RNA helicase that promotes translation initiation and regulates translation efficiency for a subset of mRNAs by binding to and unwinding the complex sequence‐structure features in the 5′ UTRs, facilitating ribosomal occupancy of mRNAs [41, 42, 46, 47]. *MYCN* mRNAs contain a structural element known as an internal ribosome entry segment (IRES) in their 5′ UTRs, responsible for increased translation levels associated with tumor growth and genotoxic stress responses during treatment [48, 49]. The 5′ UTRs in *MYCN* mRNA, including the IRES, consist of high guanine and GC‐rich sequences, potentially forming RNA structural elements recognized by DDX3X [41] (refer to Fig. S8 for predictions). Therefore, DDX3X might enhance *MYCN* translation by increasing the ribosomal occupancy of *MYCN* mRNAs. We hypothesize that *MYCN* mRNA molecules, despite their substantial production resulting from gene amplification in *MYCN*‐driven cancers, are not efficiently translated into proteins without the functional contribution of DDX3X. This potential dependency on DDX3X for efficient translation could have profound implications for MYCN‐enhanced global protein synthesis and, consequently, the growth of these cancers. This implies that targeting the interaction between DDX3X and *MYCN* mRNA could potentially hinder the excessive protein synthesis and growth associated with these cancer cells. Such an approach represents a therapeutic approach for *MYCN‐*driven cancers.

## 5 Conclusions

Our clinical observations and *in vitro* drug testing provide evidence for the anticancer activities of ceftriaxone against *MYCN*‐amplified RB and NB. Given the safe and affordable therapy as an antibiotic drug, repurposing ceftriaxone could be a novel promising strategy for treating RB and NB with high unmet needs. Our study also revealed previously unrecognized protein targets of ceftriaxone, which will merit rational drug design and future studies of RB and NB with *MYCN* amplification/overexpression.

## Conflict of interest

The authors declare no conflict of interest.

## Author contributions

PC, SR, and RK contributed to the conceptualization and design. PC, DS, SR, AS, and RK contributed to the methodology. PC, SR, VC, and RK contributed to the analysis and interpretation of the data. DR, UA, and SH provided reagents or/and clinical insights. PC and RK contributed to the writing—original draft with input from all authors, review, editing, and revising. All authors reviewed the data and approved the final version of the manuscript.

### Peer review

The peer review history for this article is available at https://www.webofscience.com/api/gateway/wos/peer‐review/10.1002/1878‐0261.13553.

## Supporting information


**Fig. S1.** Transcriptomic and Ki67‐positive cell analyses.
**Fig. S2.** Western blot analysis of MYCN and c‐MYC in retinoblastoma (RB) and Neuroblastoma (NB) cells.
**Fig. S3.** Drug–target interaction (DTI) networks.
**Fig. S4.** Structure‐based visual screening.
**Fig. S5.** Functional evaluation of NDUFA9 after ceftriaxone treatment and the response of tumor cells to cephalosporin antibiotics.
**Fig. S6.** High DDX3X expression is associated with poor prognosis.
**Fig. S7.** Translation assays.
**Fig. S8.** Prediction of G‐quadruplex and RNA‐hairpin structures which can be recognized by DDX3X in *MYCN* mRNA sequences.

**Table S1.** Ceftriaxone‐binding proteins and protein ID scores from RB170 after ceftriaxone treatment.

**Table S2.** Structure‐based visual screening of ceftriaxone‐binding proteins by molecular docking.

## Data Availability

The data that support the findings of this study are available in the [Link], [Link], [Link] of this article. The datasets generated during and/or analyzed during the current study are available in the Gene Expression Omnibus (GEO) repository (https://www.ncbi.nlm.nih.gov/geo/query/acc.cgi?acc=GSE240841).
