# Supplementary material for: Ceftriaxone exerts antitumor effects in MYCN‐driven retinoblastoma and neuroblastoma by targeting DDX3X for translation repression
Source: Mol Oncol. 2023 Nov 27;18(4):918–38. doi: 10.1002/1878-0261.13553 (PMC10994227; doi:10.1002/1878-0261.13553)
Supplement: Supplementary file 1 — Fig. S1. Transcriptomic and Ki67‐positive cell analyses. Fig. S2. Western blot analysis of MYCN and c‐MYC in retinoblastoma (RB) and Neuroblastoma (NB) cells. Fig. S3. Drug–target interaction (DTI) networks. Fig. S4. Structure‐based visual screening. Fig. S5. Functional evaluation of NDUFA9 after ceftriaxone treatment and the response of tumor cells to cephalosporin antibiotics. Fig. S6. High DDX3X expression is associated with poor prognosis. Fig. S7. Translation assays. Fig. S8. Prediction of G‐quadruplex and RNA‐hairpin structures which can be recognized by DDX3X in MYCN mRNA sequences. [file MOL2-18-918-s003.pdf]

Ceftriaxone exerts antitumor effects in *MYCN*-driven retinoblastoma and neuroblastoma by targeting DDX3X for translation repression

Supplementary Figures 1–8

Figure S1 Transcriptomic and Ki67-positive cell analyses

Figure S2 Western blot analysis of MYCN and c-MYC in retinoblastoma (RB) and Neuroblastoma (NB) cells.

Figure S3 Drug-target interaction (DTI) networks

Figure S4 Structure-based visual screening

Figure S5 Functional evaluation of NDUFA9 after ceftriaxone treatment and the response of tumor cells to cephalosporin antibiotics

Figure S6 High DDX3X expression is associated with poor prognosis

Figure S7 Translation assays

Figure S8 Prediction of G-quadruplex and RNA-hairpin structures which can be recognized by DDX3X in *MYCN* mRNA sequences

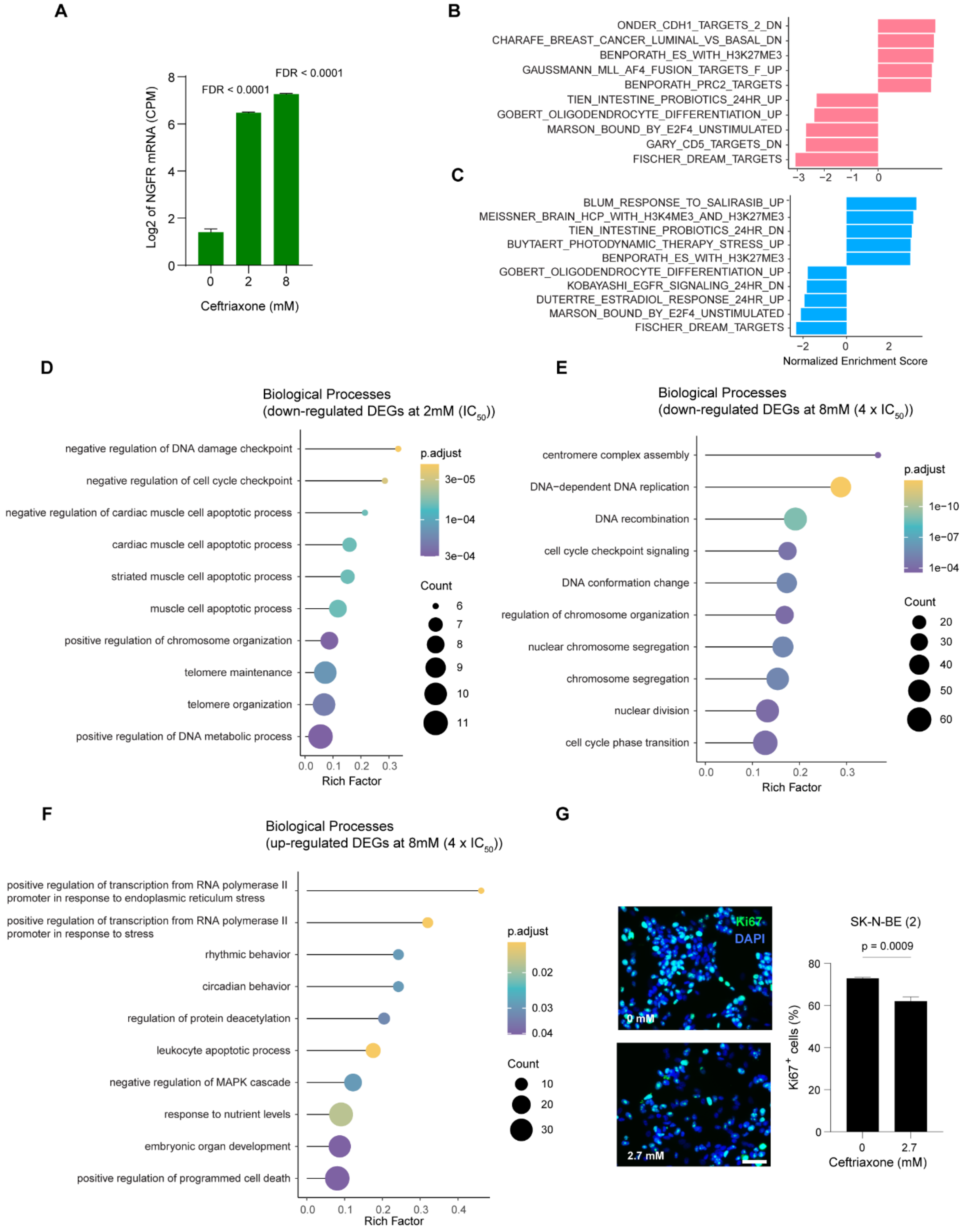

Figure S1 Transcriptomic and Ki67-positive cell analyses.

(**A**) *NGFR* mRNA levels in RB170 from RNA sequencing data

(**B, C**) The top 5 gene sets with the given positive or negative enrichment scores (NES) were enriched among differentially expressed genes (DEGs) from RB170 with

ceftriaxone (at 2 and 8 mM in B and C, respectively) compared with vehicle treatment by gene set enrichment analysis using MSigDB.

(**D**–**F**) The top 10 terms enriched among up and down-regulated DEGs after treatment with 2 mM (D) and 8 mM (E, F) ceftriaxone of RB170 by Gene Ontology analysis. Data (A–F) were derived from two independent experiments.

(**G**) Ki67 staining of SK-N-BE (2) cells, with and without drug treatment. Approximately 1300 cells were counted from 9 to 12 microscope fields (3-5 fields/experiment) for each treatment. Scale bar: 50 μm. Data were obtained from three independent experiments and analyzed using a one-way analysis of variance, followed by Dunnett's test.

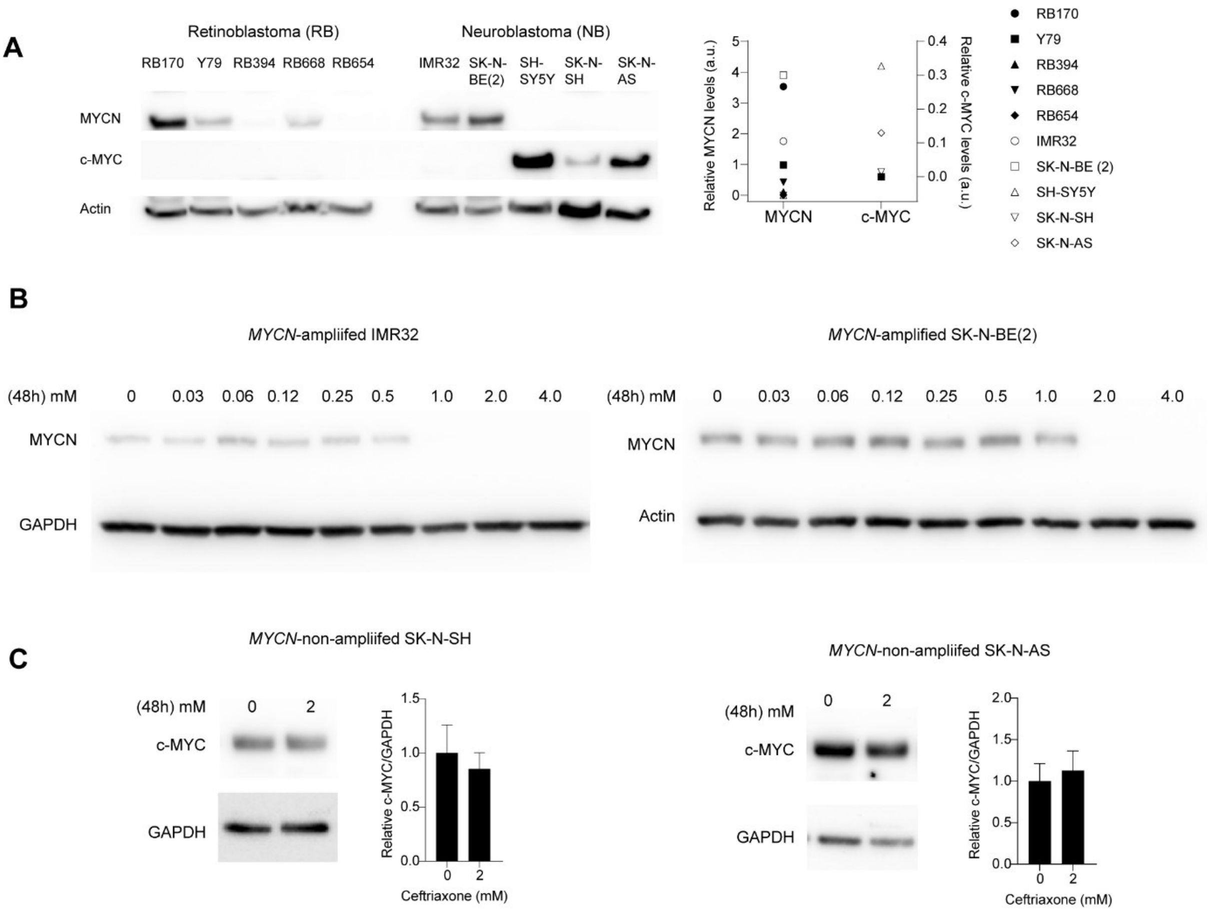

Figure S2 Western blot analysis of MYCN and c-MYC in retinoblastoma (RB) and Neuroblastoma (NB) cells.

(**A**) The expression of MYCN and c-MYC in both *MYCN*-amplified and *MYCN*-non-amplified RB and NB cells.

(**B**) MYCN expression in *MYCN*-amplified NB cells, with and without ceftriaxone treatment.

(**C**) c-MYC expression in *MYCN*-non-amplified NB cells, with and without ceftriaxone treatment. Data were obtained from three independent experiments and analyzed using a two-sample t-test.

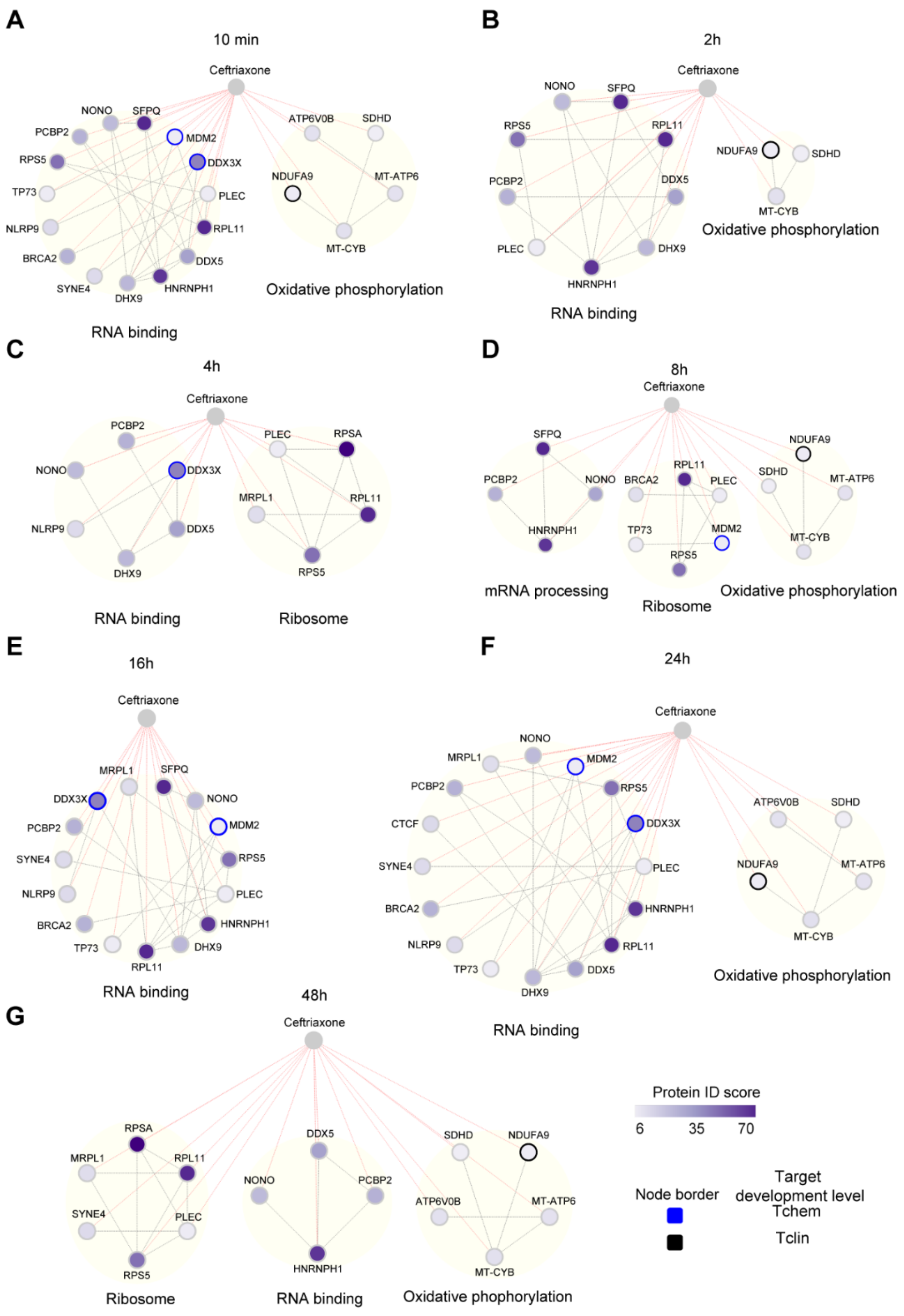

Figure S3 Drug-target interaction (DTI) networks.

(**A**–**G**) A DTI network from RB170 treated with 8 mM ceftriaxone for 10min (A), 2h (B), 4h (C), 8h (D), 16h (E), 24h (F), or 48h (G), shown with the most significant pathway (enriched from WikiPathways and KEGG Pathways). A higher protein ID score indicates a more confident match of amino acid sequences in the target protein. Tchem is a protein known to bind small molecules with high potency; Tclin is a protein *via* which approved drugs act.

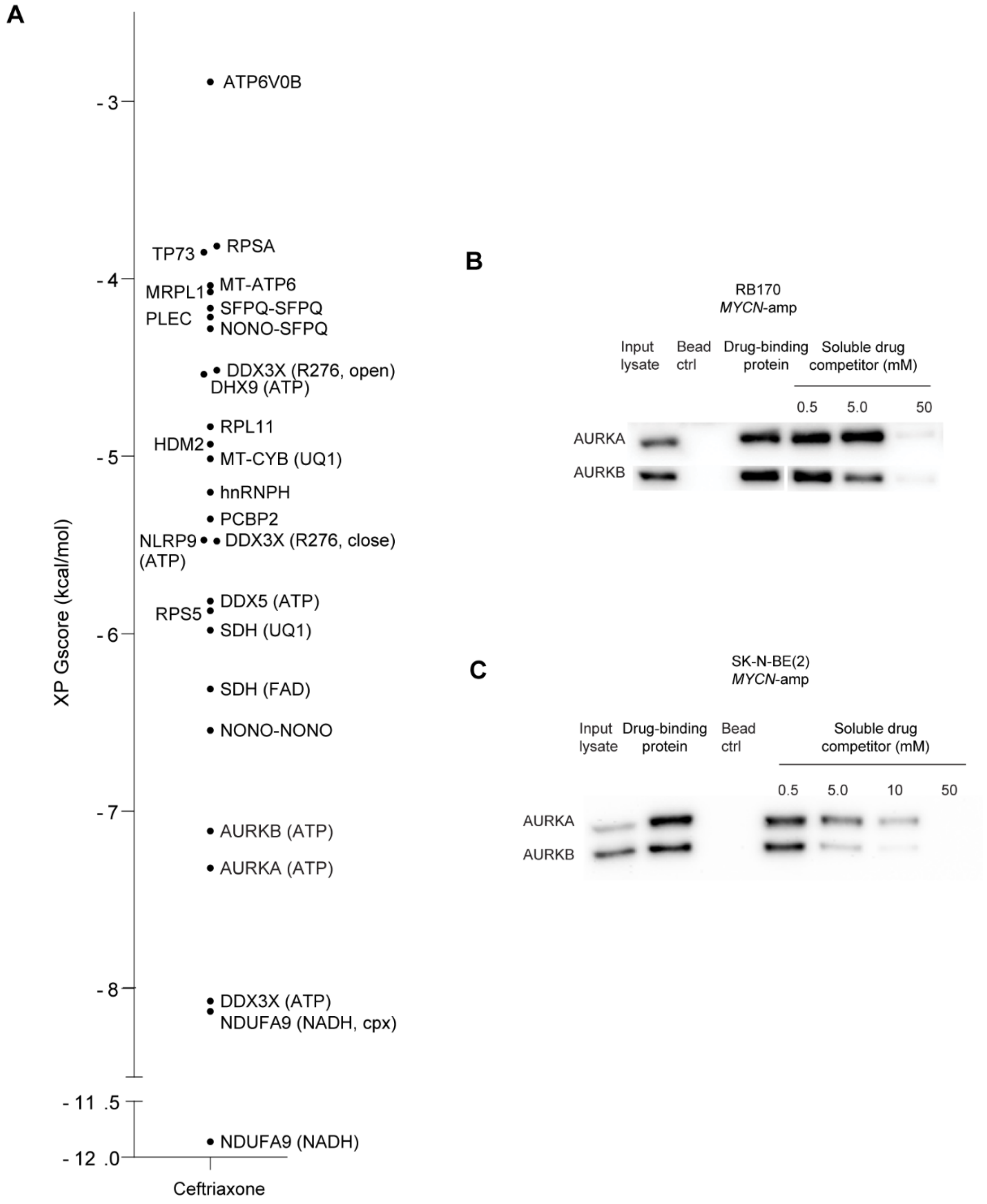

Figure S4 Structure-based visual screening.

(**A**) The diagram shows docking scores from the prediction of ceftriaxone interactions with the proteins in DTI networks. The docking sites with or without protein state details are presented in parentheses. For example, NDUFA9 (NADH, cpx) represents the docking site at the NADH-binding cleft of NDUFA9 which is in complex with the respiratory complex I. The docking sites were predicted for the proteins with unknown active sites, shown as proteins without details for docking sites. AURKA and AURKB were used as a control of ceftriaxone-binding protein.

(**B**, **C**) Affinity binding assay using ceftriaxone-conjugated sepharose beads, followed by western blotting for AURKA and AURKB in RB170 (A) and SK-N-BE (2) (B).

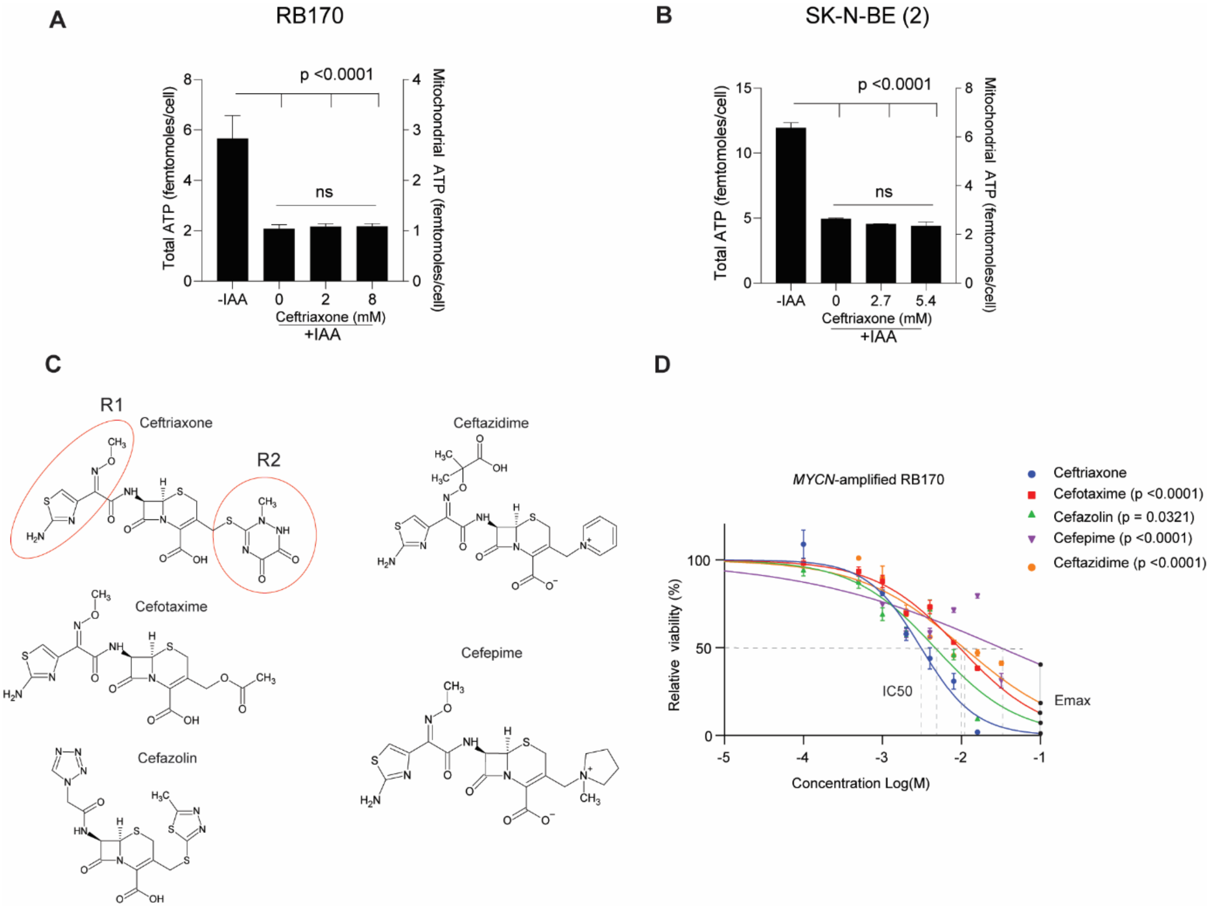

Figure S5 Functional evaluation of NDUFA9 after ceftriaxone treatment and the response of tumor cells to cephalosporin antibiotics.

(**A, B**) ATP detection assay confirms that ceftriaxone does not interfere with NDUFA9's NADH binding within the electron transport chain. Data were obtained from three independent experiments and analyzed using a one-way analysis of variance, followed by Dunnett's test. Data are shown as mean± SD, n = 3.

(**C**) Chemical structures of antibiotics from the cephalosporin family.

(**D**) Dose response curve obtained from cephem-treated RB170 organoids. The extra-sum of squares F-test was used to assess the statistical significance between the IC_{50} value of ceftriaxone and those of other cephalosporin antibiotics. IC_{50} and E_{max} represent potency and efficacy respectively.

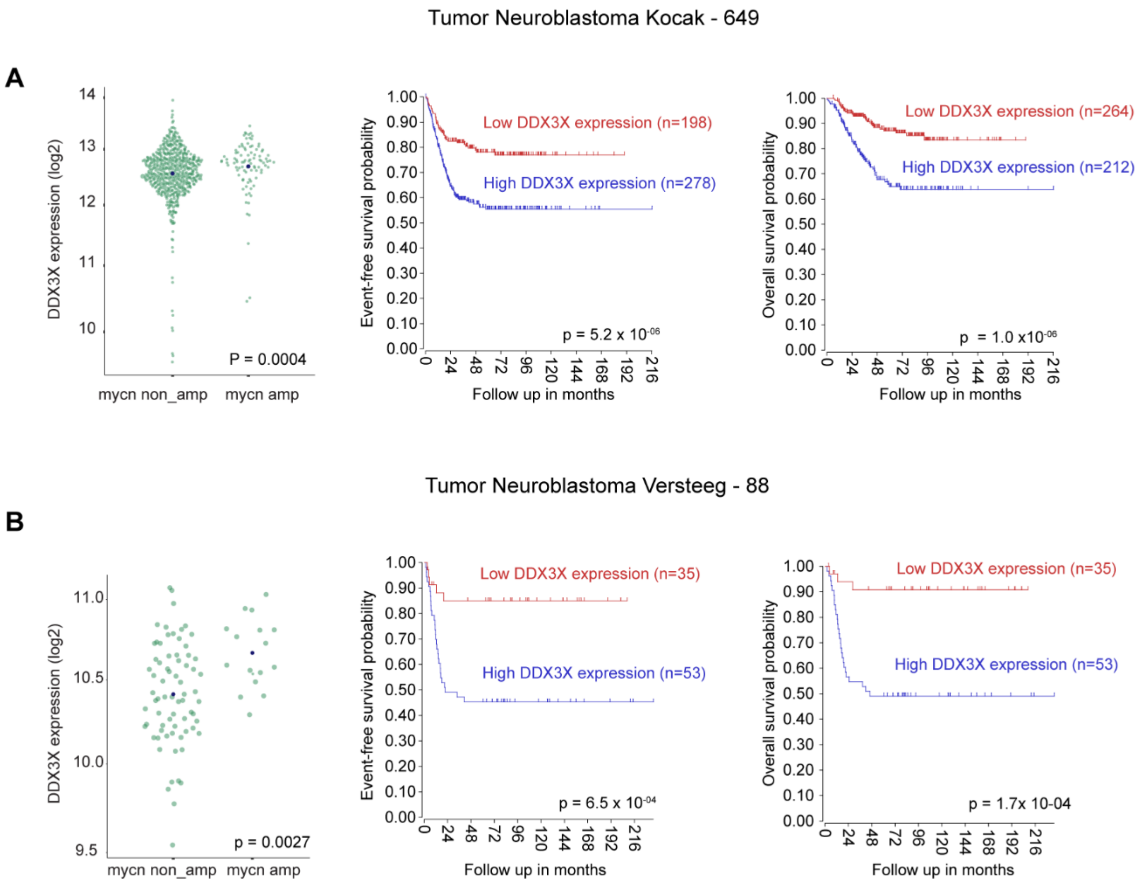

Figure S6 High *DDX3X* expression is associated with poor prognosis.

(**A**, **B**) High *DDX3X* expression levels in neuroblastoma (NB) are associated with *MYCN* amplification. Each point on the plots represents *DDX3X* levels in individual NB, and mean values are shown. Kaplan-Meier plots show the event-free and overall survival in NB patients with high (blue) and low (red) *DDX3X* mRNA expression. *DDX3X* mRNA expression cutoff was computed by Kaplan scanner to separate patient survival into two groups. The log-rank test was used for survival analysis. The analyses were performed on publicly available data (Tumor Neuroblastoma Kocak-649 (A) and Versteeg-88 (B)) from R2: Genomic Analysis and Visualization Platform (http://r2.amc.nl).

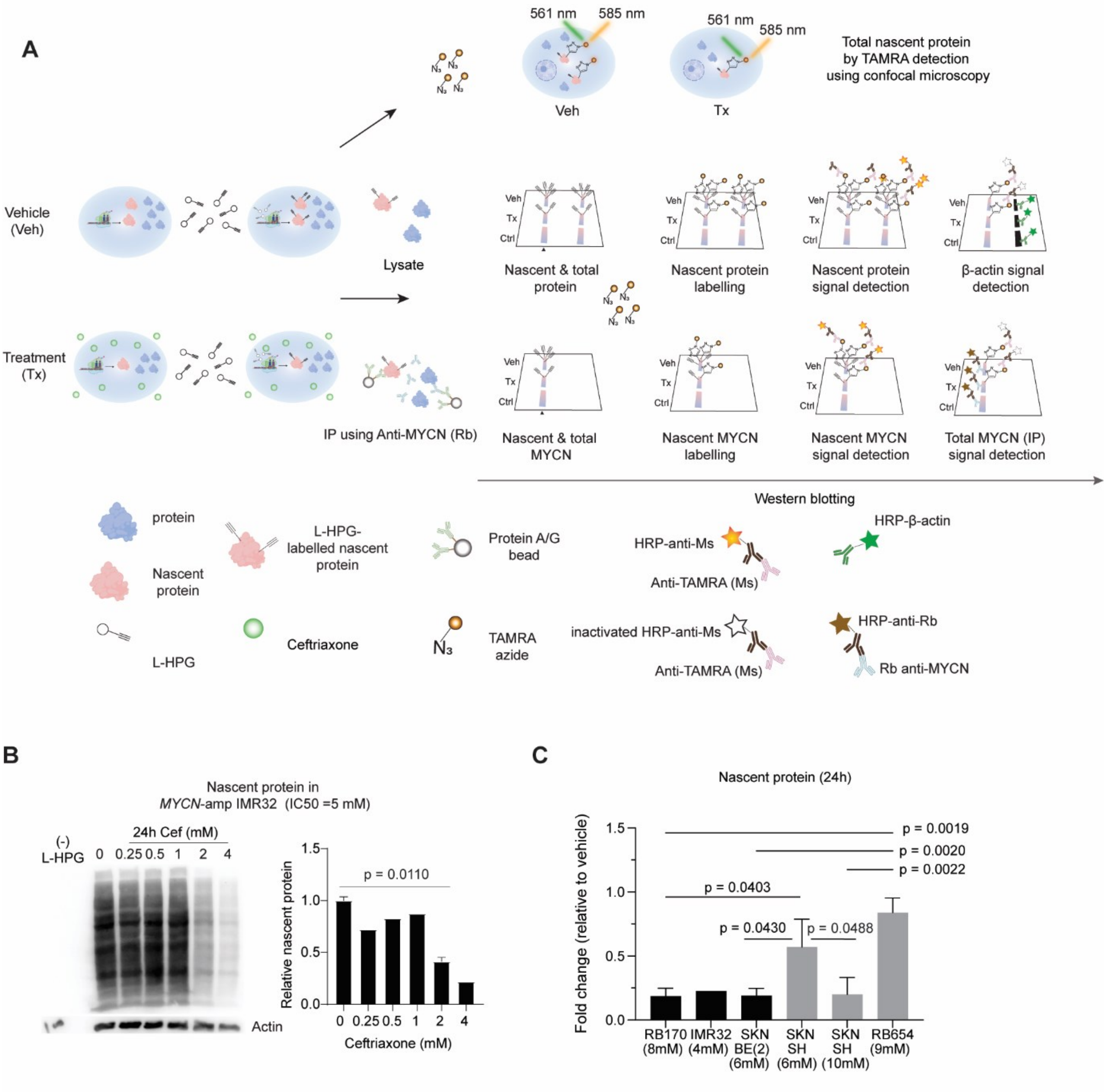

Figure S7 Translation assays.

(**A**) Schematic illustration of labeling and detecting nascent proteins and nascent MYCN using click reaction, followed by immunofluorescence or western blotting. L-homopropargylglycine, L-HPG; mouse, Ms; rabbit, Rb; IP, immunoprecipitation.

(**B**) Representative western blots show newly synthesized proteins in *MYCN*-amplified IMR32 after ceftriaxone (Cef) treatment. The normalized nascent protein is shown in bar graphs. data are shown as mean± SD, n = 2.

(**C**) Comparisons of nascent proteins from tumor cells treated with high-dose ceftriaxone. Data obtained from Figs 6A, C–E and S6B. The data were subjected to analysis using a one-way analysis of variance, followed by Tukey's test.

A

```
-300  GTCATCTGTC TGGACGCGCT GGGTGGATGC GGGGGGCTCC TGGGAACTGT GTTGGAGCCG
-240  AGCAAGCGCT AGCCAGGCGC AAGCGCGCAC AGACTGTAGC CATCCGAGGA CACCCCCGCC
-180  CCCCCGGCCC ACCCGGAGAC ACCCGCGCAG AATCGCCTCC GGATCCCCTG CAGTCGGCGG
-120  GAGTGTTGGA GGTCGGCGCC GGCCCCCGCC TTCCGCGCCC CCCACGGGAA GGAAGCACCC
 -60  CCGGTATTAA AACGAACGGG GCGGAAAGAA GCCCTCAGTC GCCGGCCGGG AGGCGAGCCG
   1  ATGCCGAGCT
```

B

Exon #	Distance from 3' Splice Site	Distance from 5' Splice Site	QGRS	G-Score
1		138	GGGTGGATGCGGGGGGCTCCTGGG	35
2	6	887	GGAGGTCGGCGCCGG	18
2	61	825	GGTATTAAAACGAACGGGGCGG	8

NCBI Reference Sequence: NM_005378

C

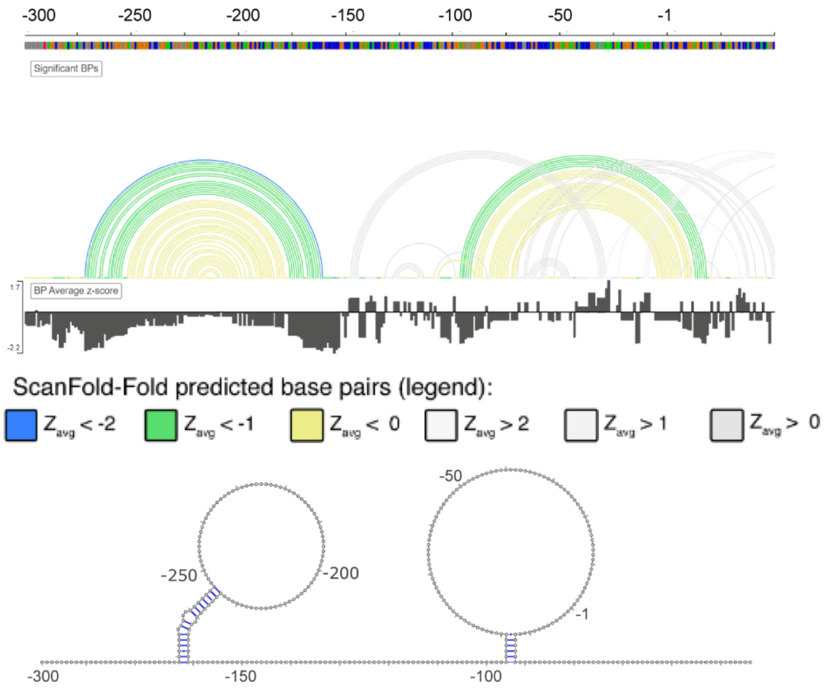

Figure S8 Prediction of G-quadruplex and RNA-hairpin structures which can be recognized by DDX3X in *MYCN* mRNA sequences.

(**A-B**) G-Rich Sequences (QGRS), highlighted in yellow, potentially form G-quadruplex in a 5' untranslated region (UTR) that contains an internal ribosome entry segment (IRES) (A). The sequences in blue between −100 and −1 are critical for *MYCN* IRES activity [49]. The positions of three QGRS regions with G-scores (B). The QGRS predicted by QGRS Mapper.

(**C**) RNA-hairpin structures predicted by ScanFold indicate significant base pairings in the critical region of *MYCN* IRES. RNA-hairpin structures with z average < -1 are shown.
